# Research on potential biomarkers of prostate cancer in Latin America and the Caribbean: a scoping review

**DOI:** 10.3389/fonc.2026.1740352

**Published:** 2026-01-30

**Authors:** Nicolas J Prada, Daniel Mendivelso-González, Sabrina Yepes, Carolay Corredor, Rafael Parra-Medina, Rodolfo Varela, Martha Lucía Serrano

**Affiliations:** 1 Grupo de Investigaciones Clínicas, Instituto Nacional de Cancerología, Bogotá, Colombia; 2 Departamento de Patología, Instituto Nacional de Cancerología, Bogotá, Colombia; 3 Grupo de Investigación en Biología del Cáncer, Instituto Nacional de Cancerología, Bogotá, Colombia; 4 Grupo Vigilancia Epidemiológica del Cáncer, Instituto Nacional de Cancerología, Bogotá, Colombia; 5 Instituto de Investigación, Fundación Universitaria de Ciencias de la Salud-FUCS, Bogotá, Colombia; 6 Departamento de Urología, Instituto Nacional de Cancerología, Bogotá, Colombia; 7 Facultad de Medicina, Universidad Nacional de Colombia, Bogotá, Colombia; 8 Departamento de Química, Facultad de Ciencias, Universidad Nacional de Colombia, Bogotá, Colombia

**Keywords:** biomarkers, Caribbean region, diagnosis, disease susceptibility, Latin America, prognosis, prostatic neoplasms, therapeutics

## Abstract

**Background:**

Latin America and the Caribbean (LAC) have higher prostate cancer (PCa) mortality rates than other regions, possibly due to disparities in detection and treatment, as well as differences in tumor biology and behavior. This scoping review aimed to identify studies conducted in LAC that evaluated potential biomarkers associated with PCa.

**Methods:**

A search was conducted in PubMed, Scopus, Embase, LILACS, and Web of Science, including original studies conducted in LAC that evaluated the presence of potential biomarkers in relation to PCa. Due to the heterogeneity of the studies, a descriptive analysis of the data was performed.

**Results:**

A total of 138 articles were included, evaluating 342 potential biomarkers across 17 countries/territories of LAC. Articles were classified into one or more of the following categories of potential biomarkers: risk of developing PCa (n=74), screening, early detection, and diagnosis (n=13), prognosis (n=48), treatment (n=10) and others (n=12). The countries with the most publications were Brazil, Mexico, and Chile.

**Conclusion:**

Most studies analyzed the relationship between various potential biomarkers and the risk of developing PCa as well as its prognosis. The majority of studies came from continental countries with lower percentages of African ancestry and lower PCa mortality rates, highlighting the need to strengthen research in LAC while improving access to healthcare. Systematic review registration:

## 1 Introduction

Cancer is currently one of the main social, economic, and public‐health challenges (1). Globally, prostate cancer (PCa) was the second most prevalent cancer among men and ranked as the fifth leading cause of cancer‐related mortality in 2022 (1). PCa incidence varies widely between regions, with the highest rates reported in North America and Oceania, and the lowest in Africa and Asia (1). Regarding mortality, this pattern shifts markedly. In regions of Africa and parts of the Caribbean, mortality rates are high despite low incidence, in contrast to highly developed areas such as Northern Europe and North America, where, despite high incidence, mortality remains low (1).

**Figure 1:**
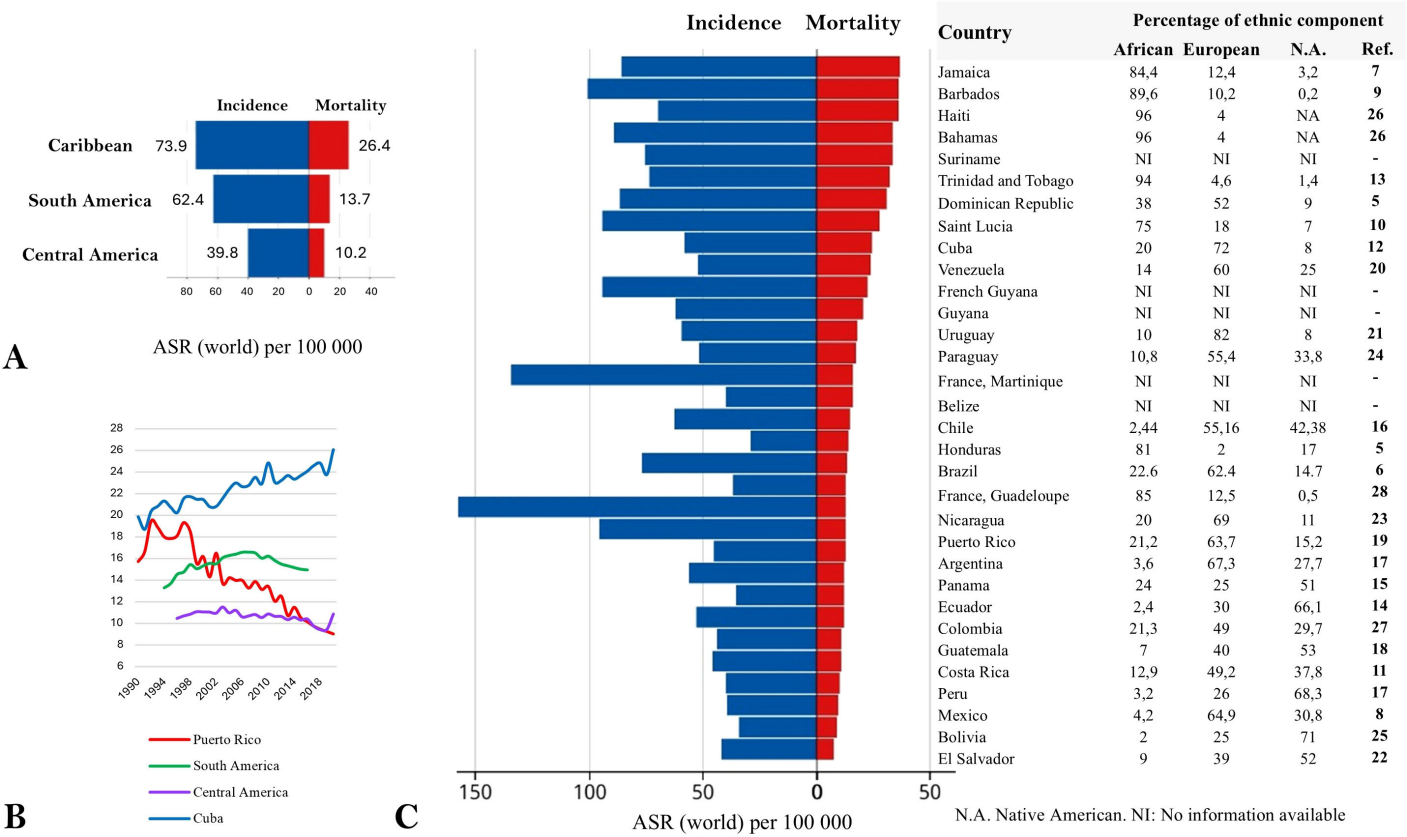
Prostate cancer incidence and mortality in Latin America and the Carribean in 2022, in relation to ethic composition. **(A)** By subrgions. **(B)** Mortality trends from 1990 to 2020 in South America, Central America, Cuba, and Puerto Rico. **(C)** By contries/territories, specifying AFRICAN, EUROPEAN, AND Native American ethnic components. Blue Barsrepresents incidence and red bars represent mortality. NA, Not data available; ASR, Age-Standardized Rate. Modified from Globocan (3).

Latin America and the Caribbean (LAC) is a region comprising 49 countries and territories (the term “territories” groups overseas and unincorporated areas) and can be subdivided into three subregions based on geographic location: Central America (8 countries), South America (12 countries and 1 territory), and the Caribbean (13 countries and 15 territories). In LAC the estimated PCa incidence and mortality rates are 58.0 and 13.9 per 100,000 men, respectively, compared with 73.5 and 8.3 in Northern America, 71.9 and 11.5 in Oceania, and 59.9 and 11.2 in Europe (2). This contrast with Asia, where incidence and mortality rates are 12.6 and 3.8, respectively (2). Within LAC, the highest PCa incidence and mortality rates were seen in the Caribbean, followed by South America, and, at the lowest level, Central America (**Figure 1A**) (2). The trend in PCa mortality between 1990 and 2020, based on Globocan data, for South America and Central America and the only two Caribbean countries with available data (Cuba and Puerto Rico), shows a striking increase in Cuba and a decrease in Puerto Rico, both Caribbean islands but with distinct geopolitical statuses, since Puerto Rico is an unincorporated territory of the United States, whereas Cuba is an independent state (**Figure 1B**) (2).

PCa incidence and mortality rates (1), and reported ethnic composition for each LAC country or overseas territory, are presented in descending order of mortality (3–26) (**Figure 1C**), where a potential association between higher mortality rates and a greater proportion of African ethnic ancestry is observed. Countries with the highest mortality were primarily Caribbean islands, except for Guadeloupe (a French overseas territory), which does not rank among them despite the fact that 85% of its population is of African descent. Interestingly, Martinique (also a French overseas territory) did not show particularly high mortality, although ethnicity data are not available for this territory. Furthermore, countries with the highest proportion of Native American ancestry, mostly located in Central America, tend to have a lower proportion of African ancestry and lower PCa mortality rates (**Figure 1C**).

The influence of ancestry on tumor biology and behavior suggests underlying differences in tumor profiles, especially in PCa. Multiple studies have reported a higher burden of PCa in men of African ancestry, linked to their West and West-Central African heritage from the transatlantic slave trade, which has been associated with earlier onset and more aggressive tumor behavior compared with men of other ancestries (27, 28). This burden is even greater in Black Caribbean populations and is also evident in the United Kingdom, where most Black men are of Afro-Caribbean or West African descent (29). These patterns likely reflect complex interactions between population-specific genetic ancestry and mixed heritage across regions, together with environmental factors, like the socioeconomic environment, that have been shown to modulate how genetic ancestry influences prostate cancer risk (28, 30). These complex interactions highlight the need for targeted strategies that extend beyond self-reported race and incorporate biomarkers, like West African ancestry–associated SNPs, which have been associated with improved prediction of biopsy positivity and clinically significant disease in African American men, highlighting the role of biomarkers in refining risk stratification and improving early detection in high-risk populations (31).

Traditionally, the term biomarker is defined as a characteristic that can be measured precisely and reproducibly, and that indicate normal or pathological biological processes, or responses to exposures or interventions (32, 33). However, due to the complexity of tumor biology, not all of these characteristics have undergone a formal validation process, and some remain at an exploratory stage. Therefore, in this review, the term potential biomarkers will be used to encompass all these findings, ranging from well-established biomarkers like PSA or PCA3 to emerging ones that are currently being studied, not only as susceptibility biomarkers but also in other areas like prognosis or treatment selection (34, 35).

LAC is characterized by high PCa mortality rates in certain areas and marked ancestral heterogeneity, with populations exhibiting varying proportions of Afro-Caribbean, Native American, and European ancestry shaped by historical processes such as colonization and the transatlantic slave trade. In this context and given the relevance of the molecular landscape for the management of this disease, this article aims to conduct a scoping review of studies on potential biomarkers related to PCa carried out in LAC countries/territories. This review will highlight the interests and advances in this field within the region, while also identifying the needs and knowledge gaps that must be addressed.

## 2 Materials and methods

This scoping review was conducted based on the PRISMA-Scr (Preferred Reporting Items for Systematic reviews and Meta-Analyses extension for Scoping Reviews) guidelines (36).

### 2.1 Search strategy

A search was conducted up to 2025 April 23 in the Medline, Embase, Scopus, LILACS and Web of Science databases, without restrictions on language or publication date. The search included terms such as “prostatic neoplasms”, “biomarker”, “Latin America” and “Caribbean”; the full search strategy is detailed in **Supplementary Table S1**. Additionally, articles identified through manual searches and from the reference lists of retrieved studies were also included.

### 2.2 Inclusion and exclusion criteria

We included original studies assessing potential biomarkers in patients with a confirmed diagnosis of PCa at any stage, residing in LAC countries/territories. The following exclusion criteria were applied: a) studies that included patients residing outside LAC and did not report disaggregated results specifically for the LAC population, b) preliminary studies whose results were part of larger studies, and c) studies with discrepancies between the methodology and the results presented.

### 2.3 Study selection and data extraction

After removing duplicates, a screening based on title and abstract was conducted. Subsequently, a full text review was performed, and the studies were classified according to their primary biomarker category, following the definitions of BEST (Biomarkers, EndpointS, and other Tools) Resource (32). The categories were adapted, for illustrative purposes, as follows: 1) related to the risk of developing PCa, 2) screening, early detection, and diagnosis, 3) prognosis, 4) treatment, and 5) others. The treatment category includes associations related to medical products, like response, predictive or safety. The others category was used for articles/biomarkers that did not directly evaluate associations with any of the previous categories. In cases where articles addressed more than one focus, they were included in all relevant categories.

Discrepancies were resolved by consensus among the authors. Information from each article was extracted into tables, including country, year of publication, authors, number of patients, study type, potential biomarker, and the technique used for its evaluation, as well as the type of association, outcome, effect measure, and/or p-value. Statistical significance was defined according to the criteria used in each article. The full data were included in the **Supplementary Tables**, while the main article contains summarized versions of the tables showing the potential biomarkers, type of association with PCa, country, and reference. These were organized according to the functional groups of the evaluated biomarkers and countries.

## 3 Results

**Figure 2:**
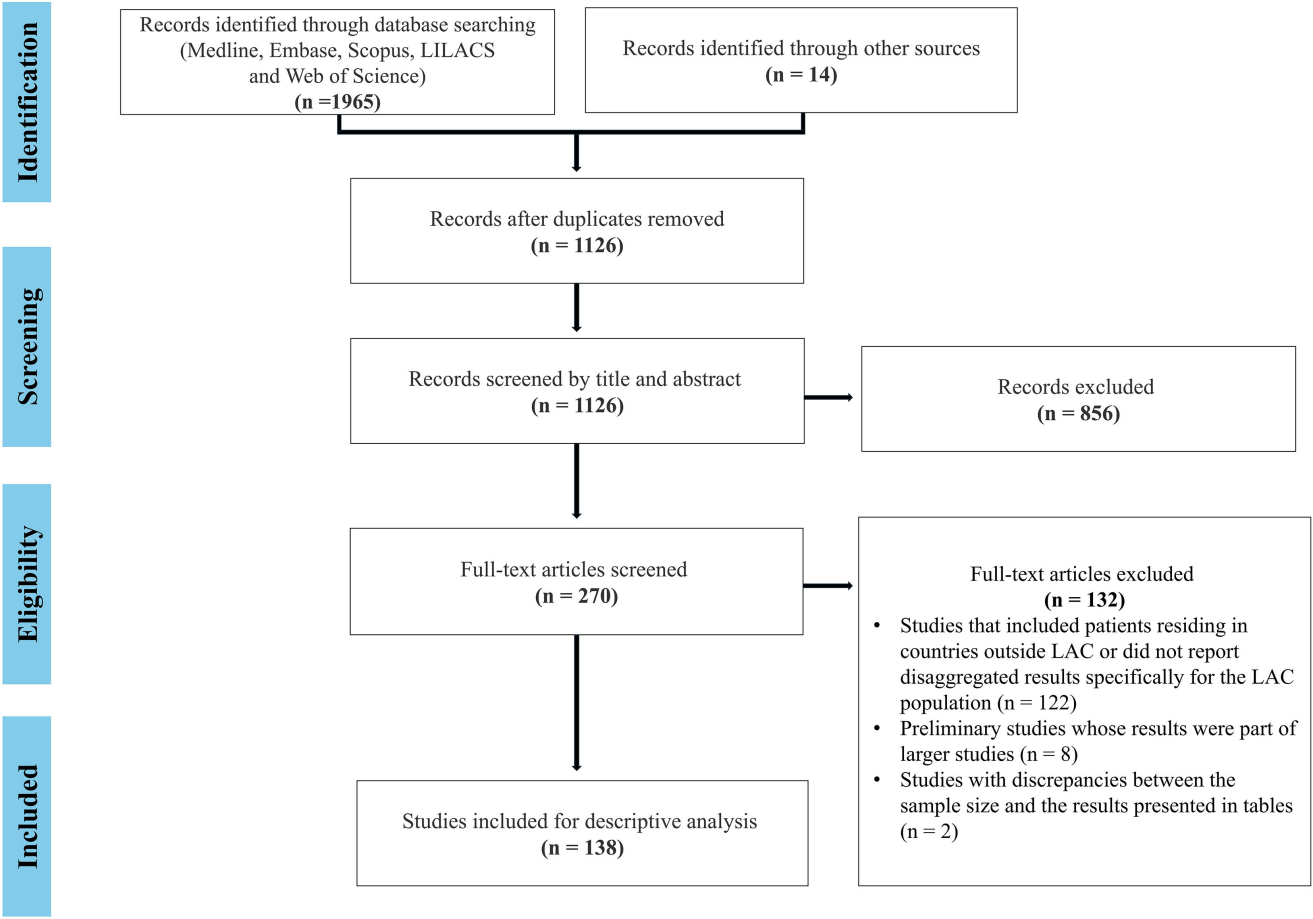
PRISMA diagram summarizing the systematic search and final selection of articles.

A total of 1979 articles were identified. After excluding duplicates and screening titles and abstracts, 270 articles underwent full-text review, and 138 of these met the inclusion criteria and were finally included. The detailed search and selection process is visually represented in the PRISMA flow diagram (**Figure 2**).

These articles were conducted in 17 LAC countries/territories: 7 from the Caribbean, 7 from South America, and 3 from Central America. The number of articles and potential biomarkers studied, classified by category and country, are presented in **Table 1**. The articles were categorized as follows: related to the risk of developing PCa (n=74); screening, early detection, and diagnosis (n=13); prognosis (n=48); treatment (n=10); and others (n=12). A total of 19 articles were classified into more than one category. Similarly, 342 potential biomarkers were reported and classified as follows: 146 related to the risk of developing PCa; 20 to screening, early detection, and diagnosis; 147 associated with prognosis; 14 linked to treatment; and 59 to the “others” group. A total of 44 biomarkers were classified into more than one category.

**Table 1:** Articles and potential biomarkers associated with prostate cancer in Latin America and the Caribbean, by country and focus category.

Country/territory	Number of articles (number of biomarkers)					
	Risk	Diagnosis	Prognosis	Treatment	Others	Total
Brazil	27 (58)	4 (12)	23 (78)	6 (11)	8 (53)	60 (193)
Mexico	12 (14)	2 (5)	5 (11)	2 (4)	3 (34)	21 (61)
Chile	3 (4)	6 (2)	6 (18)	1 (2)	1 (22)	14 (48)
Jamaica	10 (49)	–	2 (2)	–	–	11 (50)
Colombia	1 (1)	–	6 (12)	1 (1)	3 (33)	9 (44)
Puerto Rico	5 (4)	–	4 (20)	–	–	7 (22)
Argentina	–	–	2 (5)	1 (1)	2 (32)	4 (36)
Guadeloupe	4 (10)	–	1 (5)	–	–	4 (14)
Trinidad y Tobago	4 (7)	–	–	–	–	4 (7)
Ecuador	3 (4)	–	2 (5)	–	–	3 (6)
Venezuela	3 (5)	–	–	–	–	3 (5)
Peru	–	–	1 (2)	–	2 (32)	2 (32)
Martinica	1 (1)	–	1 (4)	–	1 (1)	2 (5)
Barbados	1 (1)	1 (1)	–	–	–	2 (2)
Costa Rica	–	–	–	–	1 (27)	1 (27)
Panama	–	–	–	–	1 (27)	1 (27)
Cuba	–	–	–	1 (1)	–	1 (1)
Total	74 (146)	13 (20)	48 (147)	10 (14)	12 (59)	138 (342)

The table shows the number of published articles and potential biomarkers evaluated in each country/territory, separated by category and organized in descending order, according to the total number of articles. Articles and biomarkers were classified into more than one category and/or country when applicable; therefore, the total number per row or column may be less than the direct sum of individual items.

The results are described below, grouped according to their category.

### 3.1 Potential biomarkers associated with risk of developing PCa

A total of 146 potential biomarkers associated with the risk of developing PCa were identified across 74 studies from 12 countries/territories (**Table 2**, **Supplementary Table 2**) (37–110). These were mostly case–control studies evaluating DNA polymorphisms, including one ancestry analysis, as well as expression levels measured as RNA, serum levels of proteins and vitamins, and other potential biomarkers. Most of these were SNPs and were assessed in blood and prostate tissue. The countries/territories with the highest number of publications and potential biomarkers studied were Brazil, with 27 studies and 58 biomarkers analyzed; Mexico, with 12 studies and 14 biomarkers; and Jamaica, with 10 studies and 49 biomarkers.

**Table 2:** Potential biomarkers associated with risk of developing PCa in Latin America and the Caribbean.

General function	Biomarker (association type)^Reference^	Country
Immune regulation and inflammatory response	***CCR5***: rs1799988 (Risk) (37), rs1799987 (Risk) (37, 38) ***CCR7:*** rs3136685 (Risk) (37, 38) ***CCR9:*** rs1488371 (Protector) (37) ***IRF3:*** rs2304206 (Protector) (39) ***RNASEL:*** rs1213524 (Risk) (40)	Jamaica
	***HLA-G:*** rs1707 (Risk) (41), UTR-4 haplotype (Risk) (41) ***FASL:*** rs763110 (Protector) (42)	Brazil
	***IL6ST:*** rs3729960 in HHV-8 seropositive men (Risk) (43)	Trinidad y Tobago
Xenobiotic metabolism	***NAT2:*** rs1799929 (Protector) (44), rs1208 (Protector) (44), rs1799931 (Risk) (44)	Brazil
	***GSTA1:*** rs3957357 (Protector) (45)	
	***GSTM1:*** Null genotype (Risk) (46) ***CYP1A1:*** rs4646903 (Risk) (46)	Chile
	***GSTT1:*** At least one functional allele (Risk) (47), higher copy number (Risk) (48)	Guadeloupe
Involved in prostate tissue physiology	***KLK3:*** rs266882 (Risk) (49) **PSA:** High serum levels (Risk) (50) ***NKX3-1:*** rs11781886 (Risk) (51) ***KLK2:*** High RNA levels in serum and prostate tissue (Risk) (52) ***PSMA:*** Higher expression (Risk) (53)	Brazil
	***MSMB:*** rs10993994 (Risk) (54)	Mexico
DNA repair	***APEX1:*** rs1130409 (Risk) (55) ***XPD:*** rs13181 (Risk) (56)	Brazil
	***BRCA1:*** rs1799966 (Risk) (57)	Mexico
	**DNA integrity:** DNA repair capacity with NER induction (Risk) (58, 59)	Puerto Rico
Cell adhesion and tissue remodeling	***CEACAM-1:*** Higher expression (Risk) (53) ***OPN-1:*** Higher expression (Risk) (53) **MMP-1:** High protein serum levels (Risk) (60) ***MMP1:*** rs1799750 (Risk) (61) ***MMP2:*** rs243865 (Protector) (61) ***MMP9:*** rs17576 (Risk) (61) **MMP-26:** Expression in tumoral tissue (Risk) (62) **OPNa, OPNb and OPNc isoforms:** High mRNA expression (Risk) (63) ***TSP2:*** Lower mRNA expression (Risk) (64) **TSP2:** Protein expression in stromal staining (Risk) (64)	Brazil
	***CDH1:*** rs3743674 (Risk) (65)	Jamaica
Related to sex steroid hormones	***AR:*** ≤21 CAGs repeats (Risk) (49, 66), < 19 CAGs repeats (Risk) (67), > 19 GGC repeats (Risk) (68, 69), higher expression (Risk) (53)	Brazil, Mexico, Ecuador
	***SRD5A2*:** rs523349 (Risk) (70), rs4680 (Protector) (71)	Guadeloupe, Ecuador
	***CYP19:*** > 7 CAG repeats (Risk) (71) ***COMT:*** rs4680 GG vs AA (Risk) and G allele (Risk) (72)	Guadeloupe
Endobiotic metabolism	***MTHFR:*** rs1801133 (Risk) (73) ***MTRR:*** rs1801394 (Protector) (73)	Ecuador
	***VDR:*** rs2238135 (Risk) (57), > 20 A repeats in 3’ untranslated region (Risk) (74)	Mexico, Martinique
	***PDE11A:*** Presence of sequence variations (Risk) (75)	Brazil
	**25 (OH) vitamin D:** High levels in serum (Risk) (76)	Jamaica
	***COMT*:** rs4680 (Protector) (71)	Guadeloupe
Gene expression regulation	***FTO:*** rs9939609 (Protector) (77)	Puerto Rico
	***CASC19:*** rs16901979 (Risk) (78)	Trinidad y Tobago
	**Non-coding region at 8q24.21**: rs7824364 (Risk) (79), rs6983267 (Risk) (80)	Puerto Rico, Chile
	**IGFBP-3:** high protein serum levels (Risk) (50)	Brazil
Apoptosis	***CASP3:*** rs4647603 (Risk) (51) ***CASP9:*** rs1052571 (Risk) (51)	Brazil
	***BCL-2:*** rs1801018 (Risk) (81)	Jamaica
Others	***ACE*:** Deletion of a 287-bp fragment (Risk) (82) **HPV DNA** presence in prostate tissue (Risk) (83) **Y chromosome lineage R1a** and **E1b1a/E1b1b** in Mexican Mestizo men (Risk) (84) ***VEGF:*** rs699947 (Protector) (85) ***PCA3:*** Higher expression by total number of copies (Risk) (86), higher expression (Risk) (53) ***MnSOD:*** rs4880 (87)	Mexico, Brazil
	**VEGF:** High protein expression in prostatic tissue (Risk) (88)	Chile
	**West African American Ancestry** among African American individuals (Risk) (89)	Puerto Rico

Potential biomarkers associated with the risk of developing PCa that showed statistically significant associations, grouped by functional group and by country. PCa, Prostate cancer; PSA, Prostate-specific antigen; DNA, Deoxyribonucleic acid; MMP, Matrix metalloproteinase; HPV, Human Papillomavirus; HHV-8, Human Herpesvirus-8; VEGF, Vascular endothelial growth factor.

Bold text indicates biomarker names.

The potential biomarkers were categorized into the following functional groups: immune regulation and inflammatory response (56), xenobiotic metabolism (14), gene expression regulation (13), cell adhesion and tissue remodeling (12), endobiotic metabolism (7), involved in prostate tissue physiology (7), related to sex steroid hormones (6), DNA replication and repair (3), apoptosis (4) and others (16). Biomarkers studied in more than one country included those related to the androgen receptor (AR), ribonuclease L (RNASEL), glutathione S-transferase enzymes T1, M1, and P1 (GSTM1, GSTT1, and GSTP1), cytochrome P450, 3-oxo-5α-steroid 4-dehydrogenase 2 (SRD5A2), vitamin D receptor (VDR), and the non-coding region at 8q24.21.

### 3.2 Potential biomarkers associated with PCa screening, early detection, and diagnosis

A total of 20 potential biomarkers with possible applications in the screening, early detection, and diagnosis of PCa were identified in 13 articles from four countries (**Table 3**, **Supplementary Table 3**) (111–123). These biomarkers were mainly related or compared to PSA levels as early detection biomarkers, with the aim of increasing specificity relative to PSA and thereby help determine whether a biopsy was necessary. Detailed information available from the included articles for each of these biomarkers is provided in **Supplementary Table 3**. Study designs comprised case–control, cohort, and cross-sectional studies (**Supplementary Table 3**).

**Table 3:** Potential biomarkers associated with PCa screening, early detection, and diagnosis in Latin America and the Caribbean.

General function	Biomarker ^Reference^	Country
Related to sex steroid hormones	**AR**: higher levels in PCa than BPH group in prostatic tissue samples (FFPE) (111)	Brazil
	***AR*-CAG repeat**: higher length in PCa in blood (112)	Mexico
	**microRNA related to AR expression:** **miR 27a-3p:** higher levels in PCa than BPH group in prostatic tissue samples (FFPE) (111) **miR 124:** higher levels in PCa than BPH group in prostatic tissue samples (FFPE) (111) **miR 130a:** higher levels in PCa than BPH group in prostatic tissue samples (FFPE) (111) **miR 488-3p:** higher levels in PCa than BPH group in prostatic tissue samples (FFPE) (111) **miR 506**: lower levels in PCa than BPH group in prostatic tissue samples (FFPE) (111) **EV-miR-21-5p, EV-miR-375 and EV-miR-1290-3p:** higher expression levels in the localized PCa and the mPCa group than in the control group (113) **EV-miR-200c:** higher expression levels in the mPCa group than in the control group (113)	Brazil
Inflammatory response	***COX-2:*** mRNA higher levels in blood in PCa vs. healthy men (114)	Brazil
Xenobiotic metabolism	***GSTP1:*** higher levels of promoter methylation (112)	Mexico
Cell cycle	***RASSF1A***: higher levels of promoter methylation (112) in blood sample, plasma free DNA, and USCs in PCa vs. BPH (112)	Mexico
Oxidative stress	**Mn-SOD**: overexpression of mRNA and protein in prostatic tissue of PCa vs. BPH (115) **3-NT:** overexpression of protein in prostatic tissue of PCa vs. BPH (115)	Mexico
Others	**mCPC:** higher number of detection by AMACR/P504S and PSA (116–120) **PCA3:** higher levels in urine for discriminate PCa in patients with previous negative biopsy (121)	Chile
	**PROSTest:** Increase in sensitivity and specificity for diagnosis and screening (122)	Barbados
	**Porphyrins in feces**: increased in PCa vs. without PCa (123)	Brazil

Potential biomarkers associated with PCa screening, early detection, and diagnosis, grouped by functional group and by country. PCa, Prostate cancer; BPH, Benign prostatic hyperplasia; FFPE, Formalin-fixed and paraffin-embedded; AR, Androgen receptor; AR-CAG, Androgen-receptor gene CAG repeat length; mRNA, Messenger ribonucleic acid; USCs, Urinary sediment cells; Mn-SOD, Manganese-Superoxide dismutase. 3-NT, 3-Nitrotirosine.

Bold text indicates biomarker names.

The potential biomarkers analyzed included DNA polymorphisms, gene expression levels quantified as mRNA and proteins, and metabolites, as well as methylation markers, microRNAs, and malignant primary circulating prostate cells. The analyses were based not only on blood and prostate tissue, but also on urinary sediment cells. The only related biomarkers studied in more than one country were those associated with the AR, as a protein in prostate tissue in Brazil, and as a CAG repeat length polymorphism in Mexico. The number of studies and potential biomarkers analyzed by country was as follows: Brazil, 4 studies and 12 biomarkers; Chile, 6 studies and 2 biomarkers; Mexico, 2 studies and 5 biomarkers; and Barbados, 1 study and 1 biomarker.

### 3.3 Potential biomarkers associated with PCa prognosis

A total of 48 articles (**Table 4**, **Supplementary Table 4**) (50, 58, 59, 61, 63, 64, 70, 71, 73, 74, 80, 86, 88, 109, 110, 113, 124–155) evaluating 147 potential biomarkers related to PCa prognosis were identified across 11 countries/territories. The analyses included DNA polymorphisms, protein expression levels assessed by immunohistochemistry, genotypes determined by restriction enzymes, point mutations, immune cell infiltration, DNA damage response, gene methylation, and circulating tumor DNA concentrations. These biomarkers were assessed in blood or tumor tissue.

**Table 4:** Potential biomarkers associated with PCa prognosis in Latin America and the Caribbean.

General function	Biomarker (association type)^Reference^	Country
Cell adhesion and tissue remodeling	***MMP2*:** rs1799750 (Risk) (61) ***MMP9:*** rs175768 (Protector) (61) **MMP9:** Weak (+) immunostaining in the ECM (Risk) (124) **Collagen type I:** Higher expression (Protector) (125) **Collagen type IV:** Higher expression (Protector) (125) **Collagen:** Lower deposition of collagen III compared to collagen I and II (Risk) (126) **Laminin:** Higher expression (Protector) (125) **OPNa, OPNb and OPNc isoforms:** High mRNA expression (Risk) (63) **TSP2:** Protein expression in epithelial staining (Risk) (64)	Brazil
	**E-Cadherin:** Lower expression (Risk) (127) **Beta-Catenin:** Lower expression (Risk) (127)	Chile
Related to sex steroid hormones	***AKT1 + AR:*** rs2494750+rs17302090 (Protector) (128) ***PTEN + AR:*** rs2735343+rs17302090 (Risk) (128) **Total testosterone:** Higher levels (Protector) (129)	Brazil
	***SRD5A2:*** rs9282858 (Risk) (70), rs523349 (Protector) (70)	Ecuador
	***AR:*** >20 CAGs repeats (Risk) (74)	Martinique
Involved in prostate tissue physiology	***NKX3-1:*** Higher expression (Risk) (130) ***ERG:*** Low expression (Risk) (131) ***ERG + PTEN:*** Present expression (Protector) (131)	Brazil
	**ERG:** Low expression (Risk) (132)	Colombia
Endobiotic metabolism	***AMACR:*** rs3591676 (Protector) (128), increased gene expression (Risk) (130) ***AKT1+AMACR:*** rs2494750+rs3195676 (Protector) (128) ***PTEN+AMACR:*** rs273534+rs3591676 (Risk) (128) ***PLA2G16:*** Higher expression (Protector) (133) ***PLA2G1B:*** Higher expression (Protector) (133) ***PLA2G4B:*** Higher expression (Protector) (133)	Brazil
	***ADIPOQ:*** rs266729(Risk) (134), rs1501299 (Risk) (134) ***VDR:*** TT>Tt restriction sites (Risk) (109)	Mexico
	***MTHFR:*** rs1801133 (Risk) (73)	Ecuador
	***VDR:*** > 20 ***A*** repeats in 3’ untranslated region (Risk) (74)	Martinique
	***COMT:*** rs4680 (Protector) (71)	Guadeloupe
Xenobiotic metabolism	***CYP1A1:*** *1A/*2A; *2A/*2A (Risk) (135) ***GSTM1+GSTT1+CYP1A1:*** non-null + non-null + *1A/*2A (Risk) (135) ***GSTM1:*** non-null (Risk) (135)	Chile
	***GSTP1:*** rs1695 (Risk) (136) ***GSTP1*** rs1695 + ***GSTT1*** null + ***GSTM1*** null (Risk) (136)	Argentina
	***UGT1A1:*** rs8175347 (Risk) (71)	Guadeloupe
DNA replication and repair	***TFAM:*** rs11006132(Risk) (137), rs1937(Risk) (137), rs1049432 (Risk) (137)	Mexico
	**DNA integrity:** DNA repair capacity with NER induction (59)	Puerto Rico
	***TP53*:** Presence of mutations (Risk) (138)	
Immune regulation and inflammatory response	***RNASEL:*** Asp541Glu (80) **Infiltration of B lymphocytes:** Higher infiltration (Risk) (139)	Chile
	***CXCR2:*** rs1126579 (140)	Brazil
Gene expression regulation	**Non-coding region at 8q24:** rs6983267 (Risk) (80)	Chile
Apoptosis	***BCL2:*** Lower expression (Risk) (130)	Brazil
Others	***GOLM1:*** Higher expression (Risk) (130) ***TRPM8:*** Higher expression (Risk) (130) **EV-miRNA-375-3p**, **EV-miRNA1290-3p** and a combination of both with **EV-miR-21-5p** and **EV-miR-200c-3p**: Higher expression levels (Risk) (113) **Circulating free DNA:** Higher Z-scan-derived θ values (Risk) (141) ***HIF-1:*** Lower expression (Risk) (142) ***P16:*** Lower expression (Risk) (143) ***POT1, TRF2, TPP1, TIN2, RAP1, CTC1, STN1:*** Higher expression (Risk) (144) ***TPP1, TIN2, RAP1, CTC1, STN1:*** Higher expression (Risk) (144) **Telomeres length:** Increased (risk) (144) **9p21 locus**: Deletion (Risk) (145)	Brazil
	**5hmC:** Hypomethylated genes CCDC122, NUDT15, BCCIP and KLK10 (146) **5hmC:** Hypermethylated genes PVT1, TRMT12, RPL30, UBR5, COX6C and ARMC2 (146)	Puerto Rico

Potential biomarkers associated with PCa prognosis that showed statistically significant associations, grouped by functional group and by country.

PCa, Prostate cancer; OPN, Osteopontin; TSP2, Thrombospondin 2; DNA, Deoxyribonucleic acid; 5hmC, 5-hydroxymethylcytosine.

Bold text indicates biomarker names.

The countries/territories with the highest number of publications and potential biomarkers evaluated were Brazil, with 23 studies and 78 biomarkers; Chile, with 6 studies and 18 biomarkers; Colombia, with 6 studies and 12 biomarkers; and Mexico, with 5 studies and 11 biomarkers. The biomarkers were categorized into functional groups: endobiotic metabolism (33), cell adhesion and tissue remodeling (19), DNA replication and repair (12), related to sex steroid hormones (11), immune regulation and inflammatory response (6), xenobiotic metabolism (5), involved in prostate tissue physiology (5), gene expression regulation (4), apoptosis (1) and others (39).

### 3.4 Potential biomarkers associated with PCa treatment

A total of 10 articles from 6 countries were identified, reporting on 14 potential biomarkers related to PCa treatment (**Table 5**, **Supplementary Table 5**) (156–165). Seven of these studies had an experimental focus and evaluated the application of innovative therapies in the treatment of PCa. Although the search retrieved multicenter clinical trials that included LAC centers (166, 167), these were not incorporated as they did not focus on the region or present disaggregated results for their LAC population.

**Table 5:** Potential biomarkers associated with PCa treatment in Latin America and the Caribbean.

Biomarker or target (intervention)^Reference^	Study type (population)	Findings	Country
Lymphocytes T CD3+, CD4+ and CD8+ in patients with wild-type CCR5: Increased (carboxymethyl-glucan from S. cerevisiae) (156)	Experimental study (30 patients with advanced PCa treated with ADT)	Use of carboxymethyl-glucan could stimulate cellular immune response	Brazil
DNA damage on leukocytes in peripheral blood: Reduction (carboxymethyl-glucan from S. cerevisiae) (157)	Experimental study (20 patients with advanced PCa treated with goserelin acetate)	Carboxymethyl-glucan could have a protective effect against DNA damage	
DTH reaction: Present (autologous tumor cell vaccine and Bacille Calmette-Guérin) (158)	Phase I clinical trial (11 patients with PCa)	Use of autologous tumor cell vaccine and Bacille Calmette-Guérin was safe, and it could induce cellular immune response	
CDK12 mutation (immunotherapy and DNA-damaging therapies) (159)	Case report (2 patients with mCRPC, treated with BAT & nivolumab, and radium-223 & sipuleucel-T, respectively)	The therapeutic combination could be useful for the treatment of CDK12-mutated advanced PCa	
ATM mutation (3077 + 1G>A and 8011-1G>A), SF3B1 (G742D) mutation and intermediary tumor burden: Combined immunotherapy (ipilimumab and nivolumab)	Case report (1 patient with treatment-emergent neuroendocrine PCa)	Combined immunotherapy could have therapeutic potential for the treatment of combined mutations PCa, such as ATM and SF3B1	
DNA repair defects (unspecified): Nivolumab	Phase II clinical trial (38 patients with mCRPC)	There were no statistically significant differences in studied outcomes between patients with and without DNA repair defects	
Lymphocytes T CD4+ and CD8+: Predominance of T lymphocytes over B lymphocytes infiltration and CD4:CD8 ratio of 1:4 TIA-1: Increased infiltration (Herpes virus thymidine-kinase gene + gancyclovir/valacyclovir) (160)	Phase I-II clinical trial (9 PCa patients who underwent radical prostatectomy)	Neoadjuvant AdV-tk was safe and it could stimulate anti-tumor immune response	Mexico
AR splice variant 7 (hypothetical comparation of AR signaling inhibitor vs. taxane chemotherapy) (161)	Hypothetical cost-saving analysis (N/A)	The use of AR-V7 testing could reduce costs associated with the management of mCRPC by predicting the response to AR signaling inhibitor therapy	Argentina/ Colombia/ Mexico
CD8+ IFN-γ+ T cell population: Increased DTH reaction: Increased (tumor antigen-presenting cells) (162)	Phase I clinical trial (20 patients with CRPC)	Treatment was safe, induced memory T-cell responses *in vitro* and *in vivo*	Chile
Anti-GnRH antibodies: Increased (gonadotropin-releasing hormone-based therapeutic vaccine) (163)	Phase I-II clinical trial (34 patients with advanced PCa)	Treatment induced anti-GnRH serum antibody response	Cuba

Potential biomarkers associated with PCa treatment, grouped by country. PCa, Prostate cancer; CRPC, Castration-resistant prostate cancer; mCRPC, Metastatic castration-resistant prostate cancer; BAT, Bipolar androgen therapy; DTH, Delayed-type hypersensitivity; ADT, Androgen deprivation therapy; CCR5, C-C chemokine receptor type 5; AdV-tk, Adenovirus-based vectors expressing the thymidine kinase gene; TIA-1, T-cell intracytoplasmic antigen; AR, Androgen receptor; GnRH, gonadotropin-releasing hormone.

The distribution of studies and potential biomarkers analyzed by country was as follows: Brazil, 6 studies and 11 biomarkers; Mexico, 2 studies and 4 biomarkers; Chile, 1 study and 2 biomarkers; and Argentina, Colombia and Cuba, with 1 study and 1 biomarker each. The study by Pacheco-Orozco et al. (161) was assigned to more than one country, as it was a hypothetical cost‐analysis study that evaluated the utility of the androgen receptor splice variant 7 biomarker in Argentina, Colombia and Mexico.

### 3.5 Other potential biomarkers associated with PCa

In the others category, a total of 12 articles (138, 154, 155, 168–176) were included, evaluating 59 potential biomarkers related to PCa (**Supplementary Table 6**); the majority were studies on the prevalence of germline and/or somatic mutations. The countries/territories with the highest number of publications and potential biomarkers evaluated were Brazil, with 8 studies and 53 biomarkers; Mexico, with 3 studies and 34 biomarkers; and Colombia, with 3 studies and 33 biomarkers. Among these, two multicenter studies (155, 174) stood out, evaluating the prevalence of genetic mutations in five and seven LAC countries, respectively.

## 4 Discussion

The populations of LAC have a genetic admixture of Indigenous, European, and African ancestry which, along with other factors, may influence the behavior of PCa. In this context, the aim of this study was to identify the available regional evidence in published studies on potential biomarkers associated with PCa. Across different focus categories and countries/territories, the most studied biomarkers were those related to AR, the genes *GSTM1*, *GSTT1* and *GSTP1*, and vitamin D metabolism.

Regarding AR, 5 studies from Brazil, Mexico, and Ecuador evaluated the association between CAG repeat length in the *AR* gene and the risk of PCa, three of which reported an increased risk with ≤21 CAGs (49, 66) y <19 CAGs (67). This partially aligns with the meta-analysis by Qin et al. (177), which included 51 studies from America, Africa, Asia, and Europe, and found an association between ≤20 CAGs and a higher risk of PCa. However, the other two studies did not find significant associations (99, 109). Likewise, the relationship of this biomarker with prognosis and diagnosis was also investigated; in Martinique, an association between >20 CAGs and lower PCa aggressiveness was reported (74), and in Mexico, its ability to differentiate PCa from BPH was observed (112). Similarly, other related potential biomarkers, such as AR protein expression levels in prostate tissue, and AR variant 7, were evaluated as diagnostic biomarkers and predictors of treatment response, respectively (111, 161).

With respect to glutathione S-transferases, they catalyze the conjugation of reduced glutathione to diverse compounds, enabling the detoxification of xenobiotic-derived substances, yet they can also activate oxidative metabolites with carcinogenic potential. In this review, several studies evaluated the *GSTM1* gene, and although most did not yield significant results, a study from Chile by Acevedo et al. (46) found that the Null genotype was associated with increased susceptibility to PCa. In Guadeloupe, two studies associated a higher risk with the presence of at least one functional allele of *GSTT1* (47) and with having more than two copies of this same gene (48). The meta-analysis by Gong et al. al (178)., which included studies involving Caucasian, African, and Asian individuals, found that the Null genotypes of *GSTM1* and *GSTT1* were associated with a higher risk of PCa. While Malik et al. (179) reported that the genotype Null of *GSTM1* was associated with increased risk in Asian and European populations, and genotype Null of *GSTT1* only in African populations. This may indicate differential behavior of these genes in PCa susceptibility, linked to the continental ancestry of each population.

The relationship between glutathione S-transferase genes and PCa prognosis was also studied, with the study by Acevedo et al. (135) in Chile standing out, which reported higher overall mortality in patients with the Null genotype of *GSTM1*, an association not previously described in other studies. Similarly, the study by Cotignola et al. (136) in Argentina found an association between a polymorphism of *GSTP1* and a lower biochemical recurrence-free survival. Although no significant association was found with polymorphisms of *GSTT1* and *GSTM1*, lower biochemical recurrence-free survival and higher recurrence were observed in patients when combining the genotypes of all three GST genes in multivariable models. This contrasts with the study by Nock et al. (180) in the United States, which found no differences in biochemical recurrence related to *GSTP1* gene polymorphisms in African American subjects, but did report a significant association with the Null genotype of *GSTT1*. These findings could also suggest differences in PCa prognosis based on the ancestry of the respective populations.

As for vitamin D metabolism, several potential biomarkers related to the *VDR*, such as the rs2238135 (57) polymorphism and the presence of more than 20 adenine repeats in the 3’ untranslated region of the gene, were associated with a higher risk of PCa (74); the latter was also linked to greater aggressiveness (74). These findings are consistent with studies conducted in populations from the United States (181, 182). Likewise, high serum levels of 25-hydroxyvitamin D were associated with a higher risk of PCa in Jamaica (76), which aligns with the meta-analysis by Xu et al. (183), showing a similar association in studies from Europe and the United States. This could be explained by the fact that levels of calcidiol, an intermediate metabolite generated in the liver from calcifediol, have been correlated with high levels of insulin-like growth factor 1 (184), which activates the mitogen-activated protein kinase and phosphoinositide 3-kinase pathways, and promotes cell proliferation in PCa (183, 185).

In relation to the potential biomarkers associated with PCa screening, early detection, and diagnosis, these biomarkers were primarily evaluated in relation to PSA levels ≥ 4.0 ng/mL as early detection biomarkers. This is largely due to their high sensitivity (0.90) but low specificity (0.20), which leads to a substantial number of unnecessary biopsies, as reported in reviews included in clinical practice guidelines. The primary application evaluated for these biomarkers would be to increase the specificity of PSA and thereby reduce unnecessary biopsies (186–188). Detailed information from the included articles regarding each of these biomarkers in relation to PSA is provided **in Supplementary Table 3**. Among the biomarkers included in this study, PCA3 is the only one currently addressed in clinical practice guidelines at the level of early detection; however, its usefulness in guiding biopsy decisions remains uncertain, and the supporting evidence is considered weak (186). This underscores the importance of conducting further studies to determine whether PCA3 has clinical utility in LAC.

Regarding the articles associated with treatment, there was a lack of randomized clinical trials in the final selection of studies. Although some identified studies included centers in LAC, they were excluded because they did not report results disaggregated for the LAC population (166, 167). This is particularly concerning in light of evidence showing low enrollment of individuals from minority groups in clinical trials and the resulting lack of population-specific data (189). On the other hand, although a hypothetical cost-analysis study suggested a potential cost-saving role for androgen receptor splice variant 7, direct clinical evidence in LAC is still required, as its use is not routinely recommended in clinical practice guidelines, even though some evidence suggests its utility (161, 186). Lastly, it is worth highlighting the development of trials based on innovative therapies developed within the region, which are on par with those conducted in high-income countries, such as dendritic cell-based vaccines used as neoadjuvant treatment, demonstrating the region’s potential to develop therapies tailored to its population (190, 191).

Overall, most of the studies identified were conducted individually in continental countries such as Brazil, Chile, Argentina, México, Ecuador and Colombia, which have a lower proportion of African ancestry compared to island nations like Haiti or The Bahamas (**Figure 1**). This may be explained by the lower investment in research in these insular countries/territories, with some exceptions such as Puerto Rico (192). This highlights a significant knowledge gap regarding the genetic profiles and specific characteristics of these populations, which could be linked to their higher PCa mortality rates and represent potential areas for the implementation of preventive and/or therapeutic measures. Future efforts should prioritize multicentric and collaborative studies, as well as the establishment and maintenance of shared biobanks that integrate biological, molecular, and clinical data, thereby strengthening PCa research capacity in LAC. However, while strengthening research on biomarkers is essential, these discoveries can only translate into meaningful clinical benefit if accompanied by improvements in healthcare coverage that facilitate timely diagnosis, treatment, and follow-up for the broader population.

Among the limitations of this study, the most notable is the inability to perform a meta-analysis due to the great variability of the studies found. Furthermore, well-established biomarkers may be underrepresented in the literature reviewed because they were not the main focus of current studies in the region; an example of this is the current use of commercial panels in clinical practice in LAC countries, yet no studies evaluating them were identified. Consequently, the findings do not reflect the current utilization of biomarkers and should not be used to compare patterns of use across regions; however, they highlight prevailing research trends in biomarker investigation in the region. In addition to the disparity between the countries of origin of the articles, most commonly from Brazil, Mexico and Chile, it was observed that the studies did not always include a sample representative of the ethnic composition of their country. An example is the study by Nóbrega et al. (128) in Brazil, whose sample had 85.6% European ancestry and 14.4% African ancestry, which contrasts with the values reported by de Souza et al. (3), which reported weighted average ancestry proportions of 68.1%, 19.6%, and 11.6%, for European, African, and Native American populations, respectively. Furthermore, many studies did not include an ancestry analysis of the study population and instead limited themselves to describing ethnic origin. Future studies should ensure the inclusion of representative populations and incorporate ancestry analysis to enhance the validity and regional relevance of their findings.

## 5 Conclusions

The majority of studies included in this review evaluated various types of potential biomarkers in relation to PCa risk and prognosis. The most commonly studied biomarkers were those associated with AR, glutathione S-transferase enzymes, and vitamin D metabolism. Compared to studies from other regions, some of these biomarkers showed similar expression patterns, while others differed importantly, likely reflecting genetic differences associated with continental ancestry across populations. Likewise, it was noted that most studies were conducted in continental countries of LAC with a lower proportion of African ancestry, whereas island countries/territories with higher African ancestry and higher PCa mortality rates were underrepresented. Future research in the region should address these geographic gaps and prioritize collaborative studies that provide a more comprehensive understanding of the landscape of PCa in LAC. These developments, in conjunction with appropriate improvements in access to healthcare, could facilitate the development of targeted strategies for prevention, diagnosis, and treatment for the LAC population.

## Data Availability

The original contributions presented in the study are included in the article/**Supplementary Material**. Further inquiries can be directed to the corresponding author.
