# Supplementary material for: Research on potential biomarkers of prostate cancer in Latin America and the Caribbean: a scoping review
Source: Front Oncol. 2026 Jan 30;16:1740352. doi: 10.3389/fonc.2026.1740352 (PMC12900684; doi:10.3389/fonc.2026.1740352)
Supplement: Supplementary file 1 [file Table1.docx]

***Supplementary Material***

1. **Supplementary Tables**

**Supplementary table 1. Search strategy**

| **PUBMED/SCOPUS/LILACS/Web of Science**  ("prostate cancer*" OR "prostate neoplasm*" OR "prostatic cancer*" OR "prostatic neoplasm*" OR "prostate tumor*" OR "prostatic tumor*" OR "prostate tumour*" OR "prostatic tumour*") AND ("dna" OR "rna" OR "gene*" OR "mutation*" OR "polymorphism*" OR "variant*" OR "epigenetic*" OR "gwas" OR "biomarker*" OR "genotype*" OR "molecular" OR "immunohistochem*" OR "protein*" OR "expression" OR "hybridization*" OR "fusion*" OR "deletion*" OR "amplification*" OR "insertion*" OR "translocation*" OR "duplication*" OR "inversion*" OR "germline" OR "somatic" OR "hotspot" OR "ancestr*" OR "genetic ancestr*" OR "erg" OR "etv1" OR "etv4" OR "fli1" OR "spop" OR "foxa1" OR "idh1" OR "tmprss2" OR "slc45a3" OR "elk" OR "cdk12" OR "tp53" OR "pten" OR "nkx3" OR "myc" OR "brca2" OR "atr" OR "polq" OR "neil3" OR "atm" OR "pole" OR "jag1" OR "rpa1" OR "ezh2" OR "spink1" OR "xpc" OR "exo1" OR "ercc6" OR "ncoa2" OR "stk19" OR "ddx11l1" OR "pcat1" OR "setbp1" OR "cdh1") AND ("caribbean region" OR "caribbean*" OR "latin american*" OR "latin-american*" OR "latin*" OR "hispanic* mexic*" OR "argentin*" OR "bolivia**" OR "brasil*" OR "brazil*" OR "chile*" OR "colombia*" OR "ecuador*" OR "guian*" OR "guyan*" OR "guiana francesca" OR "french guiana" OR "guyana francesa" OR "paragua*" OR "peru*" OR "surinam*" OR "urugua*" OR "venezuela*" OR "antiga e barbuda*" OR "antigua*" OR "barbuda" OR "lantigua y barbuda" OR "bahamas" OR "barbados" OR "beliz*" OR "costa ric*" OR "cuba*" OR "dominica*" OR "el salvador*" OR "granad*" OR "grenada" OR "guatemala*" OR "haiti*" OR "hondur*" OR "jamaica*" OR "nicaragua*" OR "panama*" OR "dominican republic*" OR "republica dominicana" OR "santalucia*" OR "saint lucia" OR "saocristovao e nevis" OR "san kittsnevis" OR "saint kitts" OR "nevis" OR "saovicente de grenadines" OR "san vicente y las grenadines" OR "saint vincent" OR "the grenadines" OR "trinidad et tobago" OR "trinidad y tabago" OR "trinidad" OR "tobago" OR "puerto ric*" OR "portorico" OR "guadeloup*" OR "guadalup*" OR "martiniqu*" OR "martini*" OR "saint martin*" OR "saint barthelem*" OR "america latina" OR "curaçao" OR "arub*" OR "anguilla") |
| --- |
| **EMBASE**  ('prostate cancer*':ab OR 'prostate neoplasm*':ab OR 'prostatic cancer*':ab OR 'prostatic neoplasm*':ab OR 'prostate tumor*':ab OR 'prostatic tumor*':ab OR 'prostate tumour*':ab OR 'prostatic tumour*':ab) AND ('dna':ab OR 'rna':ab OR 'gene*':ab OR 'mutation*':ab OR 'polymorphism*':ab OR 'variant*':ab OR 'epigenetic*':ab OR 'gwas':ab OR 'biomarker*':ab OR 'genotype*':ab OR 'molecular':ab OR 'immunohistochem*':ab OR 'protein*':ab OR 'expression':ab OR 'hybridization*':ab OR 'fusion*':ab OR 'deletion*':ab OR 'amplification*':ab OR 'insertion*':ab OR 'translocation*':ab OR 'duplication*':ab OR 'inversion*':ab OR 'germline':ab OR 'somatic':ab OR 'hotspot':ab OR 'ancestr*':ab OR 'genetic ancestr*':ab OR 'erg':ab OR 'etv1':ab OR 'etv4':ab OR 'fli1':ab OR 'spop':ab OR 'foxa1':ab OR 'idh1':ab OR 'tmprss2':ab OR 'slc45a3':ab OR 'elk':ab OR 'cdk12':ab OR 'tp53':ab OR 'pten':ab OR 'nkx3':ab OR 'myc':ab OR 'brca2':ab OR 'atr':ab OR 'polq':ab OR 'neil3':ab OR 'atm':ab OR 'pole':ab OR 'jag1':ab OR 'rpa1':ab OR 'ezh2':ab OR 'spink1':ab OR 'xpc':ab OR 'exo1':ab OR 'ercc6':ab OR 'ncoa2':ab OR 'stk19':ab OR 'ddx11l1':ab OR 'pcat1':ab OR 'setbp1':ab OR 'cdh1':ab) AND ('caribbean region':ab OR 'caribbean*':ab OR 'latin american*':ab OR 'latin-american*':ab OR 'latin*':ab OR 'hispanic* mexic*':ab OR 'argentin*':ab OR 'bolivia**':ab OR 'brasil*':ab OR 'brazil*':ab OR 'chile*':ab OR 'colombia*':ab OR 'ecuador*':ab OR 'guian*':ab OR 'guyan*':ab OR 'guiana francesca':ab OR 'french guiana':ab OR 'guyana francesa':ab OR 'paragua*':ab OR 'peru*':ab OR 'surinam*':ab OR 'urugua*':ab OR 'venezuela*':ab OR 'antiga e barbuda*':ab OR 'antigua*':ab OR 'barbuda':ab OR 'lantigua y barbuda':ab OR 'bahamas':ab OR 'barbados':ab OR 'beliz*':ab OR 'costa ric*':ab OR 'cuba*':ab OR 'dominica*':ab OR 'el salvador*':ab OR 'granad*':ab OR 'grenada':ab OR 'guatemala*':ab OR 'haiti*':ab OR 'hondur*':ab OR 'jamaica*':ab OR 'nicaragua*':ab OR 'panama*':ab OR 'dominican republic*':ab OR 'republica dominicana':ab OR 'santalucia*':ab OR 'saint lucia':ab OR 'saocristovao e nevis':ab OR 'san kittsnevis':ab OR 'saint kitts':ab OR 'nevis':ab OR 'saovicente de grenadines':ab OR 'san vicente y las grenadines':ab OR 'saint vincent':ab OR 'the grenadines':ab OR 'trinidad et tobago':ab OR 'trinidad y tabago':ab OR 'trinidad':ab OR 'tobago':ab OR 'puerto ric*':ab OR 'portorico':ab OR 'guadeloup*':ab OR 'guadalup*':ab OR 'martiniqu*':ab OR 'martini*':ab OR 'saint martin*':ab OR 'saint barthelem*':ab OR 'america latina':ab OR 'curaçao':ab OR 'arub*':ab OR 'anguilla':ab) |

**Supplementary table 2. Potential biomarkers associated with risk of developing PCa in Latin America and the Caribbean**

| **Country** | **Study Ref** | **Study type (number of individuals)** | **Biomarker (association type)** | **Function** | **Molecular method** |
| --- | --- | --- | --- | --- | --- |
| **Immune regulation and inflammatory response** | | | | | |
| Jamaica | Kidd et al.  2012 (50) | Case–control study  (109 PCa/102 healthy controls)^a^ | ***CCR5****:* rs1799988 GG vs. AA (Risk), rs1799987 AA vs. GG (Risk) | Expressed by T cells and macrophages, important co-receptor for viruses to enter host cells | Multiplex SNP Genotyping Method (Goldengate) |
|  | Dubey et al.  2017 (51) | Case–control study  (109 PCa/102 healthy controls) | ***CCR5****:* rs1799987 AG + GG vs. AA (Risk) |  |  |
|  | Kidd et al.  2012 (50) | Case–control study  (109 PCa/102 healthy controls)^a^ | ***CCR7:*** rs3136685 AG + GG vs. AA (Risk) | Involved in migration of memory T cells and dendritic cell maturation |  |
|  | Dubey et al.  2017 (51) | Case–control study  (109 PCa/102 healthy controls) | ***CCR7:*** rs3136685 AG + GG vs. AA (Risk) |  |  |
|  | Kidd et al.  2012 (50) | Case–control study  (109 PCa/102 healthy controls)^a^ | ***CCR9:*** rs1488371 CA + AA vs. CC (Protector) | Key regulator of thymocyte migration and maturation |  |
|  | Dubey et al.  2020 (52) | Case–control study  (162 PCa/194 healthy controls) | ***IRF3:*** rs2304206 GG vs. AA (Protector) | Promotes the transcription of interferons alpha and beta and interferon-induced genes. | TaqMan SNP genotyping by RT-PCR |
|  | Rogers et al.  2013 (78) | Case–control study  (109 PCa/102 healthy controls)^a^ | ***IRF3:*** rs968457 (NSS), rs2304206 (NSS) |  | Multiplex SNP  Genotyping Method  (Veracode) |
|  |  |  | ***IRAK4:*** rs4251467 (NSS), rs4251473 (NSS), rs4251545 (NSS) | Involved in innate immune response |  |
|  |  |  | ***TLR1:*** rs3923647 (NSS), rs4624663 (NSS), rs4833095 (NSS), rs5743595 (NSS), rs5743604 (NSS) | Identification of molecular patterns from pathogens and activation of immune response |  |
|  |  |  | ***TLR2:*** rs1898830 (NSS), rs3804099 (NSS), rs7656411 (NSS) |  |  |
|  |  |  | ***TLR4:*** rs2149356 (NSS), rs1927906 (NSS), rs1927911 (NSS), rs4986791 (NSS), rs5030710 (NSS) |  |  |
|  |  |  | ***TLR6:*** rs1039559 (NSS), rs2381289 (NSS), rs3821985 (NSS), rs5743810 (NSS), rs5743818 (NSS) |  |  |
|  |  |  | ***TLR10:*** rs10776483 (NSS), rs11096955 (NSS), rs11096957 (NSS), rs11466640 (NSS), rs4274855 (NSS) |  |  |
|  |  |  | ***TOLLIP:*** rs3168046 (NSS), rs5743867 (NSS), rs5743899 (NSS) | Interaction with Toll-like receptors signaling |  |
| Brazil | Zambra et al.  2016 (53) | Case–control study  (187 PCa/129 healthy controls) | ***HLA-G:*** rs1707 CT vs. TT (Risk), UTR-4 haplotype (Risk) | Involved in immune regulation and immune tolerance | PCR and sequencing-based genotyping |
|  | Da Silva et al.  2022 (54) | Case–control study  (123 PCa/82 healthy controls) | ***FASL:*** rs763110 TT vs. CC (Protector) | Regulation of the immune system and induction of apoptosis | PCR-RFLP |
|  | Portela et al.  2012 (79) | Case–control study  (200 PCa/185 healthy controls) | ***KIR:*** 2DL1 (NSS), 2DL2 (NSS), 2DL3 (NSS), 2DL4 (NSS), 2DL5 (NSS), 3DL1 (NSS), 3DL2 (NSS), 3DL3 (NSS), 2DS1 (NSS), 2DS2 (NSS), 2DS3 (NSS), 2DS4 (NSS), 2DP1 (NSS), 2DS5 (NSS), 3DS1 (NSS) | Regulation of the function of natural killer cells | PCR-SSP |
| Trinidad y Tobago | Henning et al.  2010 (49) | Case–control study (NA) | ***IL6ST:*** rs3729960 in HHV-8 seropositive men (Risk) | Signal transducer shared by many cytokines. | SNP analysis (method not specified) |
|  | Shea et al.  2008 (80) | Case–control study  (230 PCa/458 healthy controls) | ***ABCE1:*** -7155 polymorphism (NSS) | Role in inhibition of RNASEL | Conventional PCR and fluorescence polarization-based SNP genotyping |
|  |  |  | ***RNASEL:*** R462Q polymorphism (NSS), D541Epolymorphism (NSS) | Related to innate immunity |  |
| Jamaica | Jones et al.  2013 (48) | Case–control study  (279 PCa/535 controls) | ***RNASEL:*** rs1213524 (Risk) |  | Multiplex SNP Genotyping Method (Goldengate) |
| Venezuela | Zabala et al.  2009 (81) | Case–control study  (51 PCa/52 controls) | ***RNASEL****:* G1385A polymorphism (NSS) |  | ARMS-PCR |
|  | Pardo et al.  2019 (82) | Case–control study  (40 PCa/40 controls) | ***TNF-α:*** 238 and 308 polymorphisms (NSS) | Cytokine with key role in regulation of inflammation | PCR-RFLP |
| **Xenobiotic metabolism** | | | | | |
| Brazil | De Lima Junior et al.  2012 (56) | Case–control study  (126 PCa/74 healthy controls) | ***NAT2:*** rs1799929 (Protector), rs1208 (Protector), rs1799931 (Risk) | Activates and deactivates arylamine and hydrazine drugs and carcinogens | PCR-RFLP |
|  | D'Sa et al.  2014 (57) | Case–control study  (196 PCa/208 controls) | ***GSTA1:*** rs3957357 CT + TT vs. GG (Protector) | Detoxifies carcinogens, drugs, environmental toxins, and products of oxidative stress |  |
|  | Lima Jr et al.  2008 (84) | Case–control study  (125 PCa/100 BPH controls) | ***GSTP1:*** Missense polymorphism in nucleotide 313 [Ile 105 Val] (NSS) | Detoxification of xenobiotics, carcinogens, toxins and products of oxidative stress | PCR-SSCP |
|  |  |  | ***GSTO1:*** Missense polymorphism in nucleotide 419 [Ala 140 Asp] (NSS) |  |  |
|  |  |  | ***GSTM1:*** Present vs. Null (NSS) |  |  |
|  | De Souza et al.  2019 (83) | Case–control study  (134 PCa/134 healthy controls) | ***GSTM1:*** Non-null vs. Null (NSS) |  | qPCR |
| Venezuela | Angeli-Greaves et al.  2009 (85) | Case–control study  (50 PCa/50 BPH controls) | ***GSTM1:*** Non-null vs. Null (NSS) |  | Conventional PCR |
| Chile | Acevedo et al.  2003 (60) | Case–control study  (102 PCa/128 BPH controls) | ***GSTM1:*** Present vs. Null (Risk) |  | PCR-RFLP |
|  |  |  | ***CYP1A1:*** rs4646903 TC vs. TT (Risk) | Monooxygenase involved in drug metabolism and lipid synthesis |  |
| Brazil | Lima Jr et al.  2008 (84) | Case–control study  (125 PCa/100 BPH controls) | ***CYP1A1:*** T/C and C/C (NSS) |  | PCR-SSCP |
|  |  |  | ***GSTT1:*** Present vs. Null (NSS) | Catalyzes the binding of reduced glutathione to a variety of compounds |  |
|  | De Souza et al.  2019 (83) | Case–control study  (134 PCa/134 healthy controls) | ***GSTT1:*** Non-null vs. Null (NSS) |  | qPCR |
| Guadeloupe | Mallick et al.  2007 (58) | Case–control study  (134 PCa/134 controls) | ***GSTT1:*** At least one functional allele (Risk) |  | Multiplex PCR |
|  | Emeville et al.  2014 (59) | Case–control study  (629 PCa/622 controls) | ***GSTT1:*** Higher copy number (Risk) |  | TaqMan SNP genotyping by RT-PCR |
| Venezuela | Angeli-Greaves et al.  2009 (85) | Case–control study  (50 PCa/50 BPH controls) | ***GSTT1:*** Non-null vs. Null (NSS) |  | Conventional PCR |
| **Involved in prostate tissue physiology** | | | | | |
| Brazil | Kuasne et al.  2010 (45) | Case–control study  (170 PCa/170 controls) | ***KLK3*:** rs266882 AA vs. GG (Risk) | Protease responsible for the liquefaction of seminal coagulum | PCR-RFLP |
|  | Correa et al.  2015 (101) | Case–control study  (49 PCa/45 BPH controls) | **PSA:** High serum levels (Risk) |  | Electrochemiluminescence assay |
|  | De Souza et al.  2022 (61) | Case–control study  (283 PCa/283 healthy controls) | ***NKX3-1:*** rs11781886 CC vs. TT (Risk) | Negative regulator of epithelial cell growth in prostate tissues | TaqMan SNP genotyping by RT-PCR |
|  | Meola et al.  2006 (62) | Case–control study  (28 PCa/14 BPH controls) | ***KLK2:*** High N levels in prostate tissue (Risk), High RNA levels in serum (Risk) | Protein involved in cleaving of PSA into its active form | Multiplex semi-quantitative RT-PCR for prostate tissue.  Semi-nested RT-PCR for serum |
|  | Alonso V et al. 2009 (41) | Case–control study  (10 PCa/ 10 healthy controls) | ***PSMA :*** Higher expression (Risk) | Cell surface enzyme involved in folate metabolism and tumor progression | RT-PCR |
| Mexico | Trujillo-Cáceres et al.  2019 (65) | Case–control study  (322 PCa/628 healthy controls) | ***MSMB:*** rs10993994 TT vs. CC + CT (Risk) | Prostate secretory protein that regulates prostate growth | TaqMan SNP genotyping by RT-PCR |
| **DNA replication and repair** | | | | | |
| Brazil | Kuasne et al.  2011 (63) | Case–control study  (172 PCa/172 controls) | ***APEX1:*** rs1130409 GG + TG vs. TT (Risk) | DNA repair enzyme with apurinic/apyrimidinic activity | PCR-RFLP |
|  | Cypriano et al.  2017 (64) | Case–control study  (110 PCa/200 controls) | ***XPD:*** rs13181 AC vs. AA vs. CC (Risk) | Involved in transcription-coupled nucleotide excision repair |  |
| Puerto Rico | Matta et al.  2025 (108) | Case–control study  (71 PCa [16 mCRPCa, 31 aggressive disease and 24 with indolent tumors]  /25 controls) | **DNA repair capacity:** Percentage of DNA repair capacity with NER induction (Risk) | Impaired DNA repair capacity is linked to different types of cancer | Comet assay |
|  | Ortiz-Sánchez et al.  2022 (98) | Case–control study  (41 PCa/14 controls) | **DNA repair capacity:** Percentage of DNA repair capacity with NER induction (Risk) |  |  |
| Mexico | Martínez-Nava et al.  2020 (55) | Case–control study  (370 PCa/759 controls) | ***BRCA1:*** rs1799966 CC vs. TT (Risk) | Nuclear phosphoprotein involved in genomic stability | TaqMan SNP genotyping by RT-PCR |
| **Cell adhesion and tissue remodeling** | | | | | |
|  | Alonso et al. 2009 (41) | Case–control study  (10 PCa/ 10 healthy controls) | ***CEACAM-1:*** Higher expression (Risk) | Cell adhesion molecule associated with tumor invasion and immune modulation | RT-PCR |
|  |  |  | ***OPN-1:*** Higher expression (Risk) | Extracellular matrix protein involved in cell adhesion, immune regulation, and tissue remodeling |  |
| Brazil | El-Chaer et al.  2020 (67) | Case–control study  (20 PCa/176 BPH controls) | **MMP1:** High protein serum levels (Risk) | Involved in the breakdown of extracellular matrix proteins | TaqMan SNP genotyping by real-time PCR |
|  | Dos Reis et al.  2009 (97) | Case–control study  (100 PCa/100 controls) | ***MMP1*:** 2G/2G vs 1G/1G (Risk) | Member of the matrix metalloproteinase |  |
|  |  |  | ***MMP2:*** rs243865 CT vs. CC (Protector) |  |  |
|  |  |  | ***MMP7:*** rs11568818 GG vs AA (NSS) |  |  |
|  |  |  | ***MMP9:*** rs17576 GG vs. AA (Risk) |  |  |
|  | Silva et al.  2025 (44) | Cross-sectional study  (50 PCa/ 50 BPH/25 prostatic atrophy/25 prostatic intraepithelial neoplasia) | **MMP-26:** Expression in tumoral tissue (Risk) | Involved in breakdown of extracellular matrix | IHC |
|  | Tilli et al.  2011 (106) | Case–control study  (40 PCa/30 BPH controls) | **OPNa isoform:** High mRNA expression (Risk) | Involved in bone formation, resorption, and the expression of interferon-gamma and interleukin-12. | qRT-PCR |
|  |  |  | **OPNb isoform:** High mRNA expression (Risk) |  |  |
|  |  |  | **OPNc isoform:** High mRNA expression (Risk) |  |  |
|  | Matos et al.  2013 (107) | Case–control study  (24 PCa tissue samples/13 BPH  tissue samples)* | ***TSP2:*** Lower mRNA expression (Risk) | Inhibitor of tumor growth and angiogenesis | qRT-PCR |
|  |  | Case–control study  (204 PCa tissue samples/66 BPH  tissue samples)* | **TSP2*:*** Protein expression in stromal staining (Risk) |  | IHC |
| Jamaica | Bonilla et al.  2006 (66) | Case–control study  (89 PCa/123 controls)^a^ | ***CDH1:*** rs3743674 TC + CC vs. TT (Risk) | Calcium-dependent cell-cell adhesion protein | Direct sequencing, PCR-RFLP, and pyrosequencing |
| **Related to sex steroid hormones** | | | | | |
| Brazil | Alonso et al. 2009 (41) | Case–control study  (10 PCa/ 10 healthy controls) | ***AR:*** Higher expression (Risk) | Androgen receptor involved in prostate cell growth and hormone-driven tumor progression. | RT-PCR |
|  | Kuasne et al.  2010 (45) | Case–control study  (170 PCa/170 controls) | ***AR:*** ≤21 CAGs repeats (Risk) |  | PCR-RFLP |
|  | Neto et al.  2008 (46) | Case–control study  (49 PCa/51 healthy controls) | ***AR:*** ≤21 CAGs repeats (Risk) |  | Fluorescent PCR |
|  | Dos Santos et al.  2003 (86) | Case–control study  (133 PCa/279 healthy controls) | ***AR:*** ≤21 CAGs repeats (NSS) |  | Direct  sequencing and denaturing polyacrylamide gel electrophoresis |
|  | Biolchi et al.  2013 (47) | Case–control study  (130 PCa/88 controls) | ***AR:*** > 19 GGC repeats (Risk) |  | Fluorescent PCR |
| Mexico | Gomez et al.  2016 (68) | Case–control study  (158 PCa/326 healthy controls) | ***AR:*** > 19 GGC repeats (Risk) |  | PCR and fragment analysis by capillary electrophoresis |
|  | Patiño-García et al.  2007 (104) | Case–control study  (68 PCa/48 healthy controls) | ***AR:*** Total number of CAG repeats (NSS) |  | PCR and denaturing polyacrylamide gel electrophoresis |
| Ecuador | Paz-y-Miño et al.  2016 (69) | Case–control study  (108 PCa/226 healthy controls) | ***AR:*** < 19 CAGs repeats (Risk) |  | PCR and DNA sequencing  analysis |
|  | Paz-y-Miño et al.  2009 (100) | Case–control study  (114 PCa/144 healthy controls) | ***SRD5A2:*** rs523349 GC + CC vs. GG (Risk) | Conversion of testosterone into dihydrotestosterone | PCR-RFLP |
| Guadaloupe | Brureau et al.  2016 (99) | Case–control study  (498 PCa/565 controls) | ***SRD5A2:*** rs4680 AA vs. GG (Protector) |  | PCR and fragment analysis by capillary electrophoresis |
|  |  |  | ***CYP19:*** > 7 CAG repeats (Risk) | Catalyzes the conversion of androstenedione and testosterone to estrone and estradiol |  |
| Brazil | Dos Santos et al.  2002 (87) | Case–control study  (92 PCa/200 healthy controls) | ***CYP17:*** T-C transition in the 5’ promoter region (NSS) | Steroid biosynthesis | PCR-RFLP |
| Guadeloupe | Laurent et al.  2012 (37) | Case–control study  (709 PCa/ 723 controls) | ***CYP17:*** rs743572 (NSS) | Synthesis and catabolism of estrogens. | SNaPshot |
|  |  |  | ***COMT:*** rs4680 GG vs AA (Risk) and G allele (Risk) |  |  |
|  |  |  | ***CYP1B1:*** rs1056836 (NSS) |  |  |
|  |  |  | ***CYP19:*** rs60271534 (NSS) |  | PCR |
|  |  |  | ***UGT1A1:*** rs3064744 (NSS) |  |  |
| **Endobiotic metabolism** | | | | | |
| Ecuador | López-Cortés et al.  2013 (102) | Case–control study  (104 PCa/110 healthy controls) | ***MTHFR:*** rs1801133 CT + TT vs. CC (Risk) | Regulator of folate and homocysteine metabolism | PCR-RFLP |
|  |  |  | ***MTRR:*** rs1801394 AG + GG (Protector) | Enzyme with a key role for folate metabolism and cellular methylation |  |
| Brazil | Faucz et al.  2011 (39) | Case-control study  (50 PCa cases/ 574 healthy controls) | ***PDE11A:*** Presence of sequence variations (Risk) | Regulates intracellular signaling pathways related with hormone signaling, cell growth, and metabolism. | PCR |
| Mexico | Martínez-Nava et al.  2020 (55) | Case–control study  (370 PCa/759 controls) | ***VDR***: rs2238135 (Risk) | Transcription factor involved in mineral metabolism and immune regulation | TaqMan SNP genotyping by RT-PCR |
|  | Patiño-García et al.  2007 (104) | Case–control study  (68 PCa/48 healthy controls) | ***VDR:*** TaqI polymorphism (NSS) |  | PCR and denaturing polyacrylamide gel electrophoresis |
| Brazil | Maistro et al.  2004 (88) | Case–control study  (165 PCa/200 controls) | ***VDR:*** TaqI polymorphism (NSS) |  | PCR-RFLP |
|  |  |  | ***VDR:*** ApaI polymorphism (NSS) |  |  |
| Martinica | Veronique-Baudin et al.  2006 (96) | Case–control study  (126 PCa/127 controls) | ***VDR***: Heterozygous for long variant [> 20 A repeats in 3' untranslated region] (Risk) |  | Microsatellite analysis by PCR |
|  |  |  | ***VDR:*** Homozygous for long variant [> 20 A repeats in 3' untranslated region] (Risk) |  |  |
| Jamaica | Jackson et al.  2015 (70) | Case–control study  (224 PCa/248 controls) | **25 (OH) vitamin D:** High levels in serum (Risk) | Regulator of mineral homeostasis and musculoskeletal function | Ultra-performance liquid chromatography/  tandem mass spectrometry |
| Guadaloupe | Brureau et al.  2016 (99) | Case–control study  (498 PCa/565 controls) | ***COMT:*** rs4680 (Protector) | Conversion of catechol estrogens into inactive metabolites | PCR and fragment analysis by capillary electrophoresis |
| **Gene expression regulation** | | | | | |
| Puerto Rico | Salgado Montilla et al.  2017 (71) | Case–control study  (513 PCa/128 healthy controls) | ***FTO:*** rs9939609 AT vs. TT (Protector) | Demethylase highly expressed in lung, renal, breast and prostate cancers. | RT-PCR |
| Trinidad y Tobago | Okobia et al.  2011 (72) | Case–control study  (354 PCa/438 controls) | ***CASC19***: rs16901979 CA/AA vs. CC (Risk) | lncRNA linked to higher susceptibility to cancer. | TaqMan SNP genotyping by RT-PCR |
| Puerto Rico | Irizarry-Ramirez et al.  2015 (77) | Case–control study  (491 PCa/342 healthy controls) | **Non-coding region at 8q24.21:** rs7824364 (Risk) | Variants in this region have been linked to colon, pancreatic, and brain cancer | SNP analysis (method not specified) |
| Chile | San Francisco et al  2014 (105) | Case–control study  (83 PCa/21 healthy controls) | **Non-coding region at 8q24.21:** rs6983267 TT + GG vs. GT (Risk) |  | TaqMan SNP genotyping by RT-PCR |
| Jamaica | Murphy et al.  2012 (89) | Case–control study  (96 PCa/118 controls)^a^ | **Non-coding region at 8q24.21:** rs16900305 (NSS), rs7008482 (NSS), rs6983561 (NSS), rs16901979 (NSS), bd11934905 (NSS), rs10505477 (NSS), rs6983267 (NSS), rs7000448 (NSS), rs1447295 (NSS), rs10090154 (NSS) |  | SNP genotyping by MassARRAY |
| Brazil | Correa et al.  2015 (101) | Case–control study  (49 PCa/45 BPH controls) | **IGFBP-3:** High protein serum levels (Risk) | Regulation of mitosis | Chemiluminescence immunometric assay |
| **Apoptosis** | | | | | |
| Brazil | De Souza et al.  2022 (61) | Case–control study  (283 PCa/283 healthy controls) | ***CASP3:*** rs4647603 CT + TT (Risk) | Caspases with a major role in apoptosis and tumor suppression. | TaqMan SNP genotyping by RT-PCR |
|  |  |  | ***CASP9:*** rs1052571 GA + AA vs. GG (Risk) |  |  |
| Jamaica | Kidd et al.  2006 (74) | Case–control study  (88 PCa/126 controls)^a^ | ***BCL-2:*** rs1801018 AG/GG vs. AA (Risk) | Anti-apoptotic death regulator | SNP genotyping by pyrosequencing |
| **Others** | | | | | |
| Mexico | Sierra Diaz et al.  2009 (76) | Case–control study  (20 PCa/20 BPH controls) | ***ACE:*** Deletion of a 287-bp fragment (Risk) | Conversion of angiotensin I into angiotensin II | PCR-RFLP |
|  | Martinez-Fierro et al.  2010 (75) | Case–control study  (55 PCa/75 controls) | **HPV DNA** presence in prostate tissue (Risk) | Prevalent group of viruses associated with cervical, penile and throat cancer | Conventional PCR |
|  | Basulto-Martinez et al.  2022 (91) | Case–control study  (32 PCa/99 BPH controls) | **HPV DNA** presence in prostate tissue (NSS) |  |  |
|  | Álvarez-Topete et al.  2024 (43) | Case–control study  (152 PCa/ 372 controls) | **Y chromosome lineage R1a** in Mexican Mestizo men (Risk) | Lineages associated with cardiovascular, infertility, neurological disorders and PCa | PCR |
|  |  |  | **Y chromosome lineage E1b1a/E1b1b** in Mexican Mestizo men (Risk) |  |  |
|  | Martínez-Rizo et al.  2018 (95) | Case–control study  (211 PCa/297 BPH controls) | ***TGF-β :*** rs1800470 (NSS) | Involved in cellular proliferation, differentiation, migration and apoptosis | TaqMan SNP genotyping by RT-PCR |
|  |  |  | ***TGFBR1:*** rs334348 (NSS) | Receptor of TGF-β |  |
|  | Arámbula-Meraz E et al. (110)  2020 | Case–control study  (13 PCa/6 controls with benign prostatic diseases) | ***PCA3:*** Higher expression by total number of copies (Risk) | Marker expressed in prostate tissue and overexpressed in PCa | RT-PCR and fragment analysis |
|  | Bica et al.  2009 (40) | Case-control study  (51 PCa cases/ 372 healthy controls) | ***MnSOD:*** rs4880 CC + CT vs. TT (Risk) | Protection against oxidative damage by reactive oxygen species | PCR-RFLP |
|  | Martínez-Rizo et al.^c^  2017 (94) | Case–control study  (224 PCa/301 BPH controls) | ***VEGF:*** -1154 G/A (NSS), - 634 G/C (NSS) | Stimulates proliferation and differentiation of endothelial cells | TaqMan SNP genotyping by RT-PCR |
|  | Martinez-Fierro et al.  2013 (42) | Case–control study  (77 PCa/ 172 controls) | ***VEGF:*** rs699947 AA vs C/C (Protector) |  | PCR-RFLP |
| Chile | Acuña et al.  2013 (103) | Case–control study  (41 PCa/39 BPH controls) | **VEGF:** High protein expression in prostatic tissue (Risk) |  | Immunohistochemical staining |
| Brazil | Alonso et al. 2009 (41) | Case–control study  (10 PCa/ 10 healthy controls) | ***PCA3:*** Higher expression (Risk) | Marker expressed in prostate tissue and overexpressed in PCa | RT-PCR |
|  | Pereira et al.  2023 (38) | Cross-sectional study  (162 patients = 95 PCa, 55 without PCa, 7 with inconclusive biopsy and IHQ, and 5 with inconclusive biopsy) | **HPV DNA** (**L1 gene**) presence in prostate tissue (NSS) | Prevalent group of viruses associated with cervical, penile and throat cancer | RT-qPCR |
| Puerto Rico | Irizarry-Ramirez et al.  2017 (73) | Case–control study  (291 PCa/133 controls)^b^ | **West African American Ancestry** among African American individuals (Risk) | Correlated with PCa susceptibility | MassARRAY-based genotyping |
| Colombia | Cano et al.  2013 (92) | Case–control study  (168 PCa/140 healthy controls) | **mtDNA hypervariable segment-1:** A-U haplogroups (NSS) | mtDNA mutations have been associated with endometrial and esophageal cancer | PCR and phylogenetic analysis |
| Trinidad y Tobago | Shea et al.  2022 (93) | Case–control study  (119 PCa/223 controls) | ***ELAC2:*** S217L and A514T polymorphisms (NSS) | Maturation of mitochondrial transfer RNA | PCR-RFLP |
| Barbados | Nemesure et al.  2015 (90) | Case–control study  (641 PCa/654 controls) | **DARC** negativity (NSS) | Surface antigen of red blood cells | DARC testing (method not specified) |
| Jamaica | Elson et al.  2011 (109) | Case–control study  (81 PCa/ 81 controls) | **DARC** negativity (NSS) |  | PCR |

Potential biomarkers associated with the risk of developing PCa, grouped by functional group, country and biomarker similarity. **Notes:** a= Subgroup of Jamaican population included in the study. b= Subgroup of Puerto Rican population included in the study. c= Title refers to VEGF -2489C/T, but the abstract only includes information on VEGF polymorphisms -1154 G/A and -634 G/C. Information was collected regardless, as these SNPs were relevant to the review. **Abbreviations.** PCa: Prostate cancer. SNP: Single nucleotide polymorphisms. NSS: Not statistically significant. PCR: Polymerase chain reaction. RT-PCR: Reverse transcription polymerase chain reaction. RFLP: Restriction fragment length polymorphism. BPH: Benign prostatic hyperplasia. DNA: Deoxyribonucleic acid. SSP: Sequence-specific primers. ARMS: Amplification refractory mutation system. SSCP: Single-strand conformational polymorphism. qPCR: Quantitative polymerase chain reaction. HPV: Human Papillomavirus. DARC: Duffy antigen receptor for chemokines.

**Supplementary table 3. Potential biomarkers associated with PCa screening, early detection, and diagnosis in Latin America and the Caribbean**

| **Country** | **Study Ref.** | **Study type (number of individuals)** | **Biomarker** | **Function** | **Molecular method** | **Finding (p)** | **Biomarker association with PSA** |
| --- | --- | --- | --- | --- | --- | --- | --- |
| Brazil | Gotardelo et al. 2018 (117) | Case–control study  (9 PCa/9 controls) | **Porphyrins in faeces** | Intermediates in hem biosynthesis | Fluorescence spectroscopy | Increased levels in PCa patients (<0.001) No statistically significant correlation with PSA levels. | No statistically significant correlation with PSA levels. |
|  | Bernardes et al. 2022 (118) | Case–control study  (43 PCa/41 BPH controls) | Expression levels of **miRs (27a-3p, 124, 130a, 488-3p, and 506)** and **protein levels of AR expression** levels in prostatic tissue samples (FFPE). | miRNAs selected: Post-transcriptional gene regulation of AR. AR: Androgens signaling, PCa cells proliferation. | miRNAs: Real Time PCR  AR: Western blot | Higher levels of miR 27a-3p and AR, and  lower levels of miRs 124, 130a, 488-3p, and 506, in PCa than BPH group. | miR-27a- 3p presented a statistically significant moderate direct correlation with preoperative PSA levels in patients with CaP (r: 0.321; p value: 0.038). |
|  | Pereira et al. 2023 (119) | Case–control study  (24 PCa/25 healthy controls) | ***COX-2*** mRNA in blood | Role in prostaglandins synthesis | qRT-PCR | Higher *COX-2* expression in PCa compared to control group (p<0.045). | - |
|  | Nobrega et al.  2025 (123) | Case-control study  (30 PCa [20 with localized PCa and 10 with mPCa]  / 15 controls) | Expression of **miRNAs**  **(miR-21-5p, miR-200c-3p, miR-375-3p and miR-1290-3p**) as cf-miRNAs and EV-miRNAs | Regulation of key pathways involved in PCa development and progression | qRT-PCR | EV-miR-21-5p, EV-miR-375 and EV-miR-1290-3p had higher expression levels in the localized PCa group than in the control group  EV-miR-21-5p, EV-miR-200c, EV-miR-375 and EV-miR-1290-3p had higher expression levels in the mPCa group than in the control group | EV-miR-1290-3p (AUC 0.765, 95 %CI: 0.585–0.945; p = 0.007) and EV-4-miR-panel (AUC 0.824, 95 % CI: 0.679–0.970; p = 0.001) detected patients with PSA > 10 ng/mL |
| Mexico | Sánchez et al. 2018 (120) | Prospective cohort  (186 patients with an indication for prostate biopsy due to suspicion of cancer) | ***AR-***CAG repeat length in blood  Promoter methylation of ***GSTP1*** and ***RASSF1A*** in blood sample, plasma free DNA, and USCs. | AR: Androgens signaling, PCa cells proliferation. Glutathione-S-transferase P1: involved in elimination of genotoxic agents. Ras association domain family 1 isoform A: TSG, involved in stabilization of microtubules and cell cycle regulation | DNA sequence, sodium bisulfite conversion, quantitative methylation-specific PCR. | Significantly increase of all markers in PCa compared to BPH. Including, all of them with PSA, increase differential diagnosis accuracy. | PSA improves its differential diagnosis accuracy when combined promotor methylation of RASSF1A_plasma_ or GSTP1A_USC_ and AR-CAG repeats was performed. (AUC=0.59 to 0.70 and 0.68 respectively). Decision curve analysis (DCA) showed the utility to lessen unnecessary biopsies |
|  | Floriano-Sánchez et al.  2009 (121) | Case–control study  (27 PCa/53 BPH controls) | Manganese-Superoxide dismutase (***Mn-SOD***) and 3-Nitrotirosine (**3-NT**) genes and proteins in prostatic tissue | Antioxidants | qRT-PCR for Mn-SOD gene, IHC for Mn-SOD and 3-NT proteins. | Mn-SOD gene overexpression and Mn-SOD and 3-NT greater immunoreactivity in PCa (p<0.001) than BPH group. | No statistically significant correlation with PSA levels. |
| Chile | Ramos et al. 2013 (116) | Prospective cohort  (664 men with an indication for prostate biopsy due to suspicion of cancer) | **PCA3 i**n urine | Long non-coding RNA highly overexpresses in PCa. It regulates PRUNE2 gene. | PROGENSA PCA3 assay (based in mRNA levels) | Increase specificity for detecting PCa. Among patients with a prior negative biopsy, PCA3 specificity was 88.9% vs. 22.2% for PSA >4 ng/mL | Improve discrimination over PSA by the biomarker under study was not examined, PSA was analyzed independently and compared with PSA. Lower sensitivity of PCA3 (58%) compared with PSA>4 ng/ml (80%) but PCA3 increase specificity (87%) for detecting PCa compared with PSA (23%). Among patients with a prior negative biopsy, PCA3 specificity was 89% vs. 22% for PSA >4 ng/ml.The ROC curve analysis displayed an AUC of 0.77 for PCA3 and of 0.57 for PSA in the same group of patients (p = 0.004). |
|  | Murray et al. 2014 (111) | Prospective cohort  (559 patients undergoing biopsy) | **mCPC** detection by AMACR/P504S and PSA | Methyl-acyl-CoA racemase, involved in the β oxidation of branched-chain fatty acids and bile acid precursors | Immunocytochemistry using anti-PSA and anti-P504S. | Frequency of mCPC detection was associated with PSA levels and age. Higher number of mCPC in PCa than others. High negative predictive value 94%. | The frequency of mCPC detection increases with serum PSA >3.0ng/ml. Biopsy could be avoided if mCPC is low. |
|  | Murray et al. 2015 (112) | Prospective cohort  (664 men with an indication for prostate biopsy due to suspicion of cancer) | **mCPC** detection by P504S and PSA |  |  | Detection of mCPC showed high predictive score, sensitivity, sensibility and predictive values. PSA-AV and PSA density showed no significant difference in this population. | Improve discrimination over PSA by the biomarker under study was not examined, PSA was analyzed independently and compared with PSA. Primary CPC detection was significantly better than the two PSA derived tests (p<0.0001). AUC for PSA-AV = 0.57 (95% CI 0.53- 0.61); for PSA density = 0.62 (95% CI 0.58-0.66) and for CPC detection = 0.86 (95% CI 0.81-0.91). |
|  | Murray et al. 2015 (113) | Prospective cohort (610 men with an indication of prostate biopsy; 398<70 yrs vs. 212 >70yrs) | **mCPC** detection by P504S and PSA |  |  | mCPC detection decreased the number of prostate biopsies in fit elderly men | Improve discrimination over PSA by the biomarker under study was not examined, PSA was analyzed independently and compared with PSA. In men >70 yrs using, only PSA with a cutoff of 6.5ng/ml resulted in 50.9% of biopsies avoided but 62.2% of cancers not being detected, of which 85.7% are clinically significant. In contrast, the use of mCPC resulted in 50.5% of biopsies avoided and detection failed to detect only 2 clinically significant cancers. |
|  | Murray et al. 2015 (114) | Prospective cohort  (607 men with an indication for prostate biopsy due to suspicion of cancer) | **mCPC** detection by P504S and PSA |  |  | Primary mCPC performed better than percent free PSA and the Montreal nomogram in predicting clinically significant PCa at first prostate biopsy. | Improve discrimination over PSA by the biomarker under study was not examined, PSA was analyzed independently and compared with PSA. AUC = 0.56 for total PSA level, 0.78 for percent free PSA, 0.78 for Montreal score, and 0.84 for mCPC detection; mCPC detection had a significantly superior prediction value. Using cutoff values of percent free PSA <10%, Montreal score >50%, and ≥1 mCPC detected, mCPC detection had a higher diagnostic yield. Of the 197 cancers, 41 complied with the criteria for active surveillance; percent free PSA and the Montreal score missed a higher number of significant cancers when compared with mCPC detection. |
|  | Murray et al. 2016 (115) | Prospective cohort  (164 men who underwent a second biopsy) | **mCPC** detection by P504S and PSA |  |  | mCPC showed higher diagnostic yield than free PSA and the Chun nomogram in predicting clinically significant PCa at first prostate biopsy | Improve discrimination over PSA by the biomarker under study was not examined, PSA was analyzed independently and compared with PSA. AUCs were 0.65 for free PSA, 0.76 for the Chun score and 0.87 for CPC detection, the last having a significantly superior prediction value (p=0.01). Using cut off values of free PSA <10%, Chun score >50% and ≥1 CPC detected; CPC detection had a higher diagnostic yield. Some 4/41 cancers complied with the criteria for active surveillance, |
| Barbados | Rosin et al  2024 (122) | Prospective cohort  (509 men, two cohorts, 417 previously screened and 92 never screened) | **PROSTest** (27-marker gene circulating blood) Multigenomic liquid biopsy | Markers genes associated with PCa tumor tissue | RT-PCR/Machine learning based liquid biopsy assay | Increase in sensitivity and specificity for diagnosis and PCa screening | Improve discrimination over PSA by the biomarker under study was not examined, PSA was analyzed independently and compared with PSA. Protest AUC was 0.85±0.05 (*p*<0.0001). The sensitivity and specificity at the cut-off of 50 % was 92 % and 73 %, respectively, with a positive LR of 3.37 and a negative LR of 0.11. For PSA this was 0.71±0.1 (*p*=0.044). The sensitivity and specificity at the cut-off of 4 ng/mL was 75 % and 22 %, respectively, with a positive LR of 0.96 and a negative LR of 1.2. Combination of PSA and the PROSTest may be more accurate than PSA alone for detecting PCa |

Potential biomarkers associated with PCa screening, early detection, and diagnosis, grouped by country. **Abbreviations.** PCa: Prostate cancer. BPH: Benign prostatic hyperplasia. AR-CAG: Androgen-receptor gene CAG repeat length. USCs: Urinary sediment cells. TSG: Tumor Suppressor Gene. FFPE: Formalin-fixed and paraffin-embedded. mCPC: Malignant Primary circulating prostate cells. PSA-AV: prostate volume divided by total serum PSA. CRPC: Castration-Resistant Prostate Cancer. qRT-PCR: Real-Time Quantitative Reverse Transcription PCR. IHC: Immunohistochemistry

**Supplementary table 4. Potential biomarkers associated with PCa prognosis in Latin America and the Caribbean**

| **Country** | **Study Ref.** | **Study type (number of individuals)** | **Biomarker (association type)** | **Molecular method** | **Outcome** |
| --- | --- | --- | --- | --- | --- |
| **Cell adhesion and tissue remodeling** | | | | | |
| Brazil | dos Reis et al.  2009 (97) | Case–control study  (100 PCa/100 controls) | ***MMP1:*** 1G/2G (NSS) | TaqMan SNP genotyping by real-time PCR | Aggressiveness  (GS ≥ 7, Pathological stage pT3) |
|  |  |  | ***MMP7:*** A/G (NSS) |  |  |
|  |  |  | ***MMP2:*** rs 1799750 T vs. C *(*Risk) |  |  |
|  | Pinheiro et al.  2024 (124) | Cohort (60 PCa patients with different prognosis, adjacent tissue used as control) | **MMP2:** Immunostaining for MMP2 in the ECM (NSS) | Immunostaining of tumor tissue | Aggressiveness  (Increased TNM/ worsening prognosis group/ Advanced ISUP grade/ extraprostatic extension/ BCR) |
|  | dos Reis et al.  2009 (97) | Case–control study  (100 PCa/100 controls) | ***MMP9:*** rs 17576 8G vs. A (Protector) | TaqMan SNP genotyping by real-time PCR | Aggressiveness  (GS ≥ 7) |
|  | Pinheiro et al.  2024 (124) | Cohort (60 PCa patients with different prognosis, adjacent tissue used as control) | **MMP9:** Weak (+) immunostaining for MMP9 in the ECM (Risk) | Immunostaining of tumor tissue | Aggressiveness  (Increased TNM/ worsening prognosis group, advanced ISUP grade, extraprostatic extension, BCR) |
|  | Góes IA et al.  2024 (125) | Cohort (41 patients with localized PCa who underwent RP) | **Collagen type I**: Higher expression (Protector) | Pixels/field counting in light microscopy | Aggressiveness  (Higher GS) |
|  |  |  | **Collagen type IV**: Higher expression (Protector) |  |  |
|  |  |  | **Laminin:** Higher expression (Protector) |  |  |
|  | Pinheiro et al. 2024 (151) | Cohort study  (60 PCa patients, adjacent tissue used as control) | **Collagen**: Lower deposition of collagen III compared to collagen I and II (Risk) | Immunostaining of tumor tissue | Aggressiveness  (ISUP ≥3, ≥ T3a, PSA ≥10 ng/mL, with/without metastasis) |
|  | Tilli et al.  2011 (106) | Case–control study  (40 PCa/30 BPH controls) | **OPNa isoform:** High mRNA expression (Risk) | qRT-PCR | Aggressiveness  (GS ≥ 7 and T3 tumor stage) |
|  |  |  | **OPNb isoform:** High mRNA expression (Risk) |  |  |
|  |  |  | **OPNc isoform:** High mRNA expression (Risk) |  |  |
|  | Matos et al.  2013 (107) | Case–control study  (204 PCa tissue samples/66 BPH  tissue samples)* | **TSP2*:*** Protein expression in epithelial staining (Risk) | IHC | Aggressiveness (vascular invasion) and BCR |
|  |  |  | **TSP2*:*** Protein expression in epithelial staining (NSS) |  | Aggressiveness  (PSA > 10, GS, TNM staging, perineural invasion, capsular invasion, positive margins, seminal vesicle invasion, lymph node invasion, clinical recurrence) |
|  |  |  | **TSP2*:*** Protein expression in stromal staining (NSS) |  |  |
| Chile | Contreras et al.  2009 (126) | Case–control study  (45 PCa/15 BPH controls) | **Syndecan 1 and 2:** Lower expression *(NSS)* | Immunostaining of tumor tissue | Aggressiveness  (GS ≥ 7) |
|  |  |  | **E-Cadherin:** Lower expression (Risk) |  |  |
|  |  |  | **Beta-Catenin:** Lower expression (Risk) |  |  |
| **Related to sex steroid hormones** | | | | | |
| Brazil | Nóbrega et al.  2020 (127) | Case–control study  (277 PCa/277 healthy controls) | ***AKT1 + AR:*** rs2494750 + rs17302090 CC+A (Protector) | RT-PCR | Aggressiveness  (Seminal vesicle invasion) |
|  |  |  | ***AKT1 + AR:*** rs2494750 + rs17302090 CC+A (Protector) |  | Aggressiveness  (Bilaterality) |
|  |  |  | ***PTEN + AR:*** rs2735343 + rs17302090 CG+ CG+A (Risk) |  | Aggressiveness  (Extracapsular extension) |
|  | Correa et al.  2015 (101) | Case–control study  (49 PCa/45 BPH controls) | **Total testosterone:** Higher levels (NSS) | Chemiluminescence immunometric assay | Aggressiveness  (GS ≥ 7) |
|  | Melão et al.  2024 (142) | Retrospective study  (212 PCa patients) | **Total testosterone:** Higher levels (Protector) | NA | Aggressiveness  (biopsy ISUP grade) and BCR |
|  |  |  | ***AR:*** Relative expression AR/B2M (NSS) | qPCR | BCR |
|  |  |  | ***ARV7:*** Positive expression (NSS) |  |  |
| Ecuador | Paz-y-Miño et al.  2009 (100) | Case–control study  (114 PCa/144 healthy controls) | ***SRD5A2:*** rs9282858 V89L AA vs. AT + TT (Risk) | PCR-RFLP | Aggressiveness  (Higher pTNM stage/ elevated GS grade) |
|  |  |  | ***SRD5A2:*** rs523349 V89L A49T VV vs. VL + LL (Protector) |  | Aggressiveness  (Progression to a higher tumor stage) |
| Martinica | Veronique-Baudin et al.  2006 (96) | Case–control study  (126 PCa/127 controls) | ***SRD5A2:*** SRD5A2-S/ SRD5A2-S (NSS) | Microsatellite analysis by PCR | Aggressiveness  (GS > 7) |
|  |  |  | ***AR:*** >20 CAGs repeats (Protector) |  | Aggressiveness  (Advanced PCa and GS > 7) |
| Mexico | Patiño-García et al.  2007 (104) | Case–control study  (68 PCa/48 healthy controls) | ***AR:*** Number of CAG repeats (NSS) | PCR and denaturing polyacrylamide gel electrophoresis | Aggressiveness  (GS > 7) |
| Guadeloupe | Brureau et al.  2016 (99) | Case–control study  (498 PCa/565 controls) | ***CYP19:*** rs60271534 >7 of TTTA tandem repeats (NSS) | PCR and fragment analysis by capillary electrophoresis | Aggressiveness  (GS > 7) |
|  |  |  | ***CYP17:*** rs743572 TT vs. A (NSS) |  |  |
| **Involved in prostate tissue physiology** | | | | | |
| Brazil | Souza et al. 2020 (129) | Cohort study (60 patients with localized PCa who underwent RP) | ***NKX3-1:*** Higher expression (Risk) | RT-PCR and liquid biopsy | Aggressiveness  (GS >7) |
|  | Morais et al.  2019 (130) | Cohort (119 Patients with localized PCa who underwent RP) | ***ERG:*** Low expression (Risk) | Immunostaining of tumor tissue | Aggressiveness  (GS >6) |
|  | Eguchi et al.  2014 (137) | Case–control study (98 PCa patients/ 27 healthy controls) | ***ERG:*** Positive expression (NSS) | RT-PCR | BCR |
|  |  |  | ***TMPRSS2-ERG fusion:*** Positive expression (NSS) | Immunostaining of tumor tissue | BCR |
|  | Morais et al.  2019 (130) | Cohort study  (119 patients with localized PCa who underwent RP) | **ERG + PTEN:** Present expression (Protector) | Immunostaining of tumor tissue | Aggressiveness  (Higher GS and higher GG) |
|  | Yorioka et al. 2025 (152) | Cohort study  (101 patients with low risk PCa) | **PTEN**: Expression loss in tumoral tissue (NSS) | Immunostaining of tumor tissue | BCR |
|  |  |  | **ERG:** Positive expression in tumoral tissue (NSS) |  |  |
| Colombia | Acosta-Vega et al.  2023 (131) | Cohort study (78 patients with localized PCa who underwent RP) | ***ERG:*** Low expression (Risk) | RNA sequencing | BCR-FS |
|  | Segura-Moreno et al.  2022 (138) | Cohort study (20 PCa patients who underwent RP) | ***ERG:*** Higher expression (NSS) | RT-PCR | BCR |
|  |  |  | ***NKX3-1:*** Low expression (NSS) |  | BCR |
|  | Montero-Ovalle et al.  2023 (139) | Cohort study (112 patients with localized PCa who underwent RP) | ***TMPRSS2-ERG fusion:*** Positive expression (NSS) | FISH | BCR |
|  | Segura-Moreno et al.  2022 (138) | Cohort study (20 PCa patients who underwent RP) |  | RT-PCR | BCR |
| **Endobiotic metabolism** | | | | | |
| Brazil | Souza et al.  2020 (129) | Cohort study (60 patients with localized PCa who underwent RP) | ***AMACR:*** Increased gene expression (Risk) | RT-PCR and liquid biopsy | Aggressiveness  (Increased extracapsular extension) |
|  | Nóbrega et al.  2020 (127) | Case–control study  (277 PCa/277 healthy controls) | ***AMACR:*** rs3591676 GA GA+AA (Protector) | RT-PCR | Aggressiveness  (Seminal vesicle invasion) |
|  |  |  | ***AKT1+AMACR:*** rs2494750+rs3195676 CC+GA (Protector) |  |  |
|  |  |  | ***PTEN+AMACR:*** rs273534 + rs3591676 (CG+GG (Risk) |  | Aggressiveness  (Extracapsular extension) |
|  | Recuero et al.  2024 (145) | Cohort study  (108 PCa cases and 6 controls) | ***PLA2G16:*** Higher expression (Protector) | RT-PCR | Aggressiveness  (Increased TNM) |
|  |  |  | ***PLA2G1B:*** Higher expression (Protector) |  |  |
|  |  |  | ***PLA2G4B:*** Higher expression (Protector) |  | Aggressiveness  (Increased TNM, Higher GS)  and BCRFS |
|  |  |  | ***PLA2G1B, PLA2G2A, PLA2G2D, PLA2G4A, PLA2G4B, PLA2G4C, PLA2G4D, PLA2G4E, PLA2G4F, PLA2G6, PLA2G7, PLA2G16, PNPLA1, PNPLA2:*** Higher expression (NSS) |  | Aggressiveness  (Increased TNM, Higher GS) and OS |
| Ecuador | López-Cortés et al.  2013 (102) | Case–control study  (104 PCa/110 healthy controls) | ***MTHFR:*** rs1801133 C/C vs. C/T + T/T (Risk) | PCR-RFLP | Aggressiveness  (GS 7–10) |
|  |  |  | ***MTHFR:*** rs1801131 A/A vs. A/C + C/C (NSS) |  |  |
|  |  |  | ***MTR:*** rs1805087 AA vs. A/G + G/G (NSS) |  |  |
|  |  |  | ***MTRR:*** rs1801394 AA vs. A/G + G/G (NSS) |  |  |
| Mexico | Canto et al.  2017 (133) | Cross-sectional study  (257 overweight or obese patients with PCa) | ***ADIPOQ:*** rs266729 C>G (Risk) | RT-PCR | Aggressiveness  (Higher risk on D’Amico classification) |
|  |  |  | ***ADIPOQ:*** rs1501299 276G/T (Risk) |  |  |
|  |  |  | **ADIPOQ:** rs7665116 T>A (NSS) |  |  |
|  | Patiño-García et al.  2007 (104) | Case–control study  (68 PCa/48 healthy controls) | ***VDR:*** TT>Tt restriction sites (Risk) | PCR and denaturing polyacrylamide gel electrophoresis | Aggressiveness  (GS > 7) |
| Colombia | Montero-Ovalle et al.  2023 (139) | Cohort study  (112 patients with localized PCa who underwent RP) | ***IDH1:*** Point mutations (NSS) | FISH | BCR |
|  | Segura-Moreno et al.  2022 (138) | Cohort study  (20 PCa patients who underwent RP) | ***SPINK:*** Higher expression (NSS) | RT-PCR | BCR |
| Martinica | Veronique-Baudin et al.  2006 (96) | Case–control study  (126 PCa/127 controls) | ***VDR***: Heterozygous for long variant [> 20 A repeats in 3' untranslated region] (Risk) | Conventional PCR | Aggressiveness  (Advanced PCa and GS ≥ 7) |
|  |  |  | ***VDR:*** Homozygous for long variant [> 20 A repeats in 3' untranslated region] (Risk) |  | Aggressiveness  (Advanced PCa and GS ≥ 7) |
| Guadeloupe | Brureau et al.  2016 (99) | Case–control study  (498 PCa/565 controls) | ***COMT:*** rs4680 A [Val] vs. G [Met] (Protector) | PCR and fragment analysis by capillary electrophoresis | Aggressiveness  (GS 4+3 or >7 and advanced clinical stage) |
| **Xenobiotic metabolism** | | | | | |
| Guadeloupe | Brureau et al.  2016 (99) | Case–control study  (498 PCa/565 controls) | ***CYP1B1:*** rs1056836 G [Val] vs. A [Leu] (NSS) | PCR and fragment analysis by capillary electrophoresis | Aggressiveness  (GS >7 and advanced clinical stage) |
| Chile | Acevedo et al.  2014 (128) | Cohort study  (260 PCa patients) | ***CYP1A1***, *1A /*2A;*2A/*2A (Risk) | PCR-RFLP | Increased overall mortality |
|  |  |  | **GSTM1*:*** *N*on-null vs. Null (Risk) |  |  |
|  |  |  | ***GSTM1 +GSTT1+CYP1A:*** non-null+non-null+*1A/*2A (Risk) |  |  |
|  |  |  | ***GSTT1:*** Null vs. Non-null (NSS) |  |  |
| Argentina | Cotignola et al.  2013 (132) | Cohort study  (105 PCa patients) | ***GSTT1:*** Present vs. Null (NSS) | Multiplex-PCR | Shorter BRFS |
|  |  |  | **GSTM1:** Present vs. Null (NSS) |  |  |
|  |  |  | **GSTP1*:*** rs1695 AA + AG vs. GG (Risk) | PCR-RFLP |  |
|  |  |  | ***GSTP1 + GSTT1+ GSTM1:*** rs1695 c.313 A4G + null + null (Risk) | PCR-RFLP and Multiplex-PCR |  |
| Guadeloupe | Brureau et al.  2016 (99) | Case–control study  (498 PCa/565 controls) | ***UGT1A1:*** rs8175347 >6 of TA repeats on TATA box (Risk) | PCR and fragment analysis by capillary electrophoresis | Aggressiveness  (GS > 7) |
| **DNA replication and repair** | | | | | |
| Argentina,  Colombia, Peru,  Chile,  Mexico | Angel et al.  2024 (155) | Prospective, cross-sectional, multicenter study  (349 metastatic PCa patients  [Argentina (n=164), Colombia (n=147), Peru (n=15), Chile (n=14), Mexico (n=9)] | **BRCA1, BRCA2:** Presence of mutations (NSS) in mCRPCa patients | Next generation sequencing | Progression-free survival |
| Colombia | Montero-Ovalle et al.  2023 (139) | Cohort study  (112 patients with localized PCa who underwent RP) | ***FOXA1:*** Point mutations (NSS) | FISH | BCR |
|  |  |  | ***SPOP:*** Point mutations (NSS) |  |  |
|  | Segura-Moreno et al.  2022 (138) | Cohort study  (20 PCa patients who underwent RP) |  | RT-PCR |  |
|  |  |  | ***EZH2:*** Higher expression (NSS) |  |  |
| Puerto Rico | Ortiz-Sánchez et al.  2022 (98) | Case–control study  (41 PCa/14 controls) | **DNA repair capacity:** Percentage of DNA repair capacity with NER induction (NSS) | Comet assay | Aggressiveness  (GG 3, GG 4, GG5) |
|  | Matta et al.  2025 (108) | Case–control study  (71 PCa [16 mCRPCa, 31 with aggressive disease and 24 with indolent tumors]  /25 controls) | **DNA repair capacity:** Percentage of DNA repair capacity with NER induction (Risk) |  | Aggressiveness  (GS 4+3 and ≥8) |
| Mexico | Granados et al.  2016 (134) | Cross-sectional study  (257 overweight or obese patients with PCa) | ***TFAM:*** rs11006132 (Risk) | TaqMan SNP genotyping by real-time PCR | Aggressiveness  (D'Amico classification of intermediate or high risk) |
|  |  |  | ***TFAM:*** rs1937 (Risk) |  |  |
|  |  |  | ***TFAM:*** rs1049432 A-G-G (Risk), G-C-T (NSS), G-G-T (NSS), G-C-T (NSS), A-G-G (NSS) |  |  |
| Brazil | Freitas et al.  2024 (154) | Prospective study  (89 PCa patients) | ***TP53*:** Presence of mutations (Risk) | Next-generation sequencing | Aggressiveness  (GS ≥ 8) |
| **Immune regulation and inflammatory response** | | | | | |
| Chile | San Francisco et al  2014 (105) | Case-control study  (83 PCa/ 21 healthy controls) | ***RNASEL:*** Asp541Glu 8C/C (Risk) | TaqMan SNP genotyping by real-time PCR | Aggressiveness  (Preoperative PSA) |
|  | Rodríguez et al.  2023 (136) | Cohort study  (70 PCa patients) | **Infiltration of T lymphocytes:** Higher infiltrate (NSS) | Immunostaining of tumor tissue | Aggressiveness  (Higher GS) |
|  |  |  | **Infiltration of B lymphocytes:** Higher infiltrate (Risk) |  |  |
| Brazil | Franz et al.  2017 (135) | Case–control study  (175 PCa/ 201 healthy controls) | ***CXCR2:*** rs1126579 CT vs. CC (Protector) | PCR-SSP | Aggressiveness  (Clinical stage T3-T4) |
|  |  |  | ***IL-8:*** rs4073 251 T/A (NSS) |  |  |
| Colombia | Parra-Medina et al. (141) | Cohort study  (23 PCa patients) | **CCR7*:*** Positive expression (NSS) | Immunostaining of tumor tissue | Aggressiveness  (Higher GS and nodal stage) |
| **Gene expression regulation** | | | | | |
| Brazil | Correa et al.  2015 (101) | Case–control study  (49 PCa/45 BPH controls) | **Serum IGF-I:** Higher expression (NSS) | Chemiluminescence immunometric assay | Aggressiveness  (GS ≥ 7) |
|  |  |  | ***IGFBP-3:*** Higher expression (NSS) |  |  |
| Chile | San Francisco et al  2014 (105) | Case–control study  (83 PCa/ 21 healthy controls) | **Non-coding region at 8q24:** rs6983267 8G/G vs. G/T-T/T (Risk) | TaqMan SNP genotyping by real-time PCR | Aggressiveness  (Preoperative PSA) |
| **Apoptosis** | | | | | |
| Brazil | Souza et al.  2020 (129) | Cohort study  (60 PCa patients who underwent RP) | ***BCL2:*** Lower expression (Risk) | RT-PCR and liquid biopsy | Aggressiveness  (Bilateral tumor) |
| **Others** | | | | | |
| Brazil | Souza et al.  2020 (129) | Cohort study  (60 PCa patients who underwent RP) | ***GOLM1:*** Higher expression (Risk) | RT-PCR and liquid biopsy | Higher risk of developing aggressive PCa  (GS >7) |
|  |  |  | ***TRPM8:*** Higher expression (Risk) |  |  |
|  |  |  | ***OR51E2:*** Higher expression (NSS) |  | Aggressiveness  (Several histopathological features) |
|  |  |  | ***SIM2:*** Higher expression (NSS) |  |  |
|  |  |  | ***PCA3:*** Higher expression (NSS) |  |  |
|  | Nobrega et al.  2025 (123) | Case-control study  (30 PCa [20 with localized PCa and 10 with mPCa]  / 15 controls) | **EV-miRNA-375-3p:** Higher expression levels (Risk)  **EV-miRNA1290-3p:** Higher expression levels (Risk) | qRT-PCR | Aggressiveness  (ISUP ≥ 3) |
|  |  |  | **EV-miRNA1290-3p:** Higher expression levels (Risk)  **EV-miR-21-5p + EV-miR-200c-3p + EVmiR-375-3p + EV-miR-1290-3p:** Higher expression levels (Risk) |  | Aggressiveness  (PSA > 10 ng/mL) |
|  | Alves et al  2025 (143) | Cross-sectional study  (14 PCa patients) | **Circulating free DNA:** Higher Z-scan-derived θ values (Risk) | Z-scan technique (nonlinear spectrophotometry) | Clinical relapse at 6 months |
|  |  |  | **Circulating free DNA:** Higher Z-scan-derived θ values (NSS) |  | Aggressiveness  (Higher PSA, GS >6) and  BCR at 6 months |
|  | Febronio et al.  2025 (146) | Cohort (57 patients with localized PCa) | **HIF-1:** Higher expression (NSS) | Immunostaining of tumor tissue | Aggressiveness  (Higher GS) |
|  |  |  | **HIF-1:** Lower expression (Risk) |  | Aggressiveness  (Higher pT) |
|  | Cavalcante et al.  2025 (147) | Cohort  (488 PCa patients who underwent RP) | **P16:** Lower expression (Risk) | Immunostaining of tumor tissue | BCR |
|  | Dos Santos et al.  2024 (148) | Cohort (81 PCa patients who underwent RP /10 controls with hyperplastic tissue) | **POT1, TRF2, TPP1, TIN2, RAP1, CTC1, STN1:** Higher expression (Risk) | RT-PCR | Aggressiveness  (Higher pT) |
|  |  |  | **TPP1, TIN2, RAP1, CTC1, STN1:** Higher expression (Risk) |  | BCR |
|  |  |  | **Telomeres length:** Increased (risk) | qRT-PCR | Aggressiveness  (Higher TNM stage, higher GS) and  BCR |
|  | De Barros et al.  2017 (150) | Cohort (111 patients with localized PCa who underwent RP) | **9p21 locus**: Deletion (Risk) | FISH | BCR |
|  |  |  | **9p21 locus:** Deletion (NSS) |  | Aggressiveness  (Higher GS, Higher stage) |
| Puerto Rico | Ruiz-Deya et al.  2021 (140) | Cohort study  (24 patients with PCa) | ***RREB1, FAM71F2, JMJD1C, COL5A3, RAE1, GABRQ:*** Hypermethylated (NSS) | Illumina 850K DNA methylation platform | Aggressiveness  (Higher GS) |
|  |  |  | ***COL9A2, FAM179A, SLC17A2, PDE10A, PLEKHS1, TNNI2, OR51A4, RNF169, SPNS2, ADAMTSL5, CYP4F12:*** Hypomethylated, (NSS) |  |  |
|  | Acuña et al.  2013 (103) | Case–control study  (41 PCa patients/ 39 BPH patients) | **VEGF [VEGF-A, VEGF-B, VEGF-C, VEGF-D]:** Marking percentage (NSS) | Immunostaining of tumor tissue | BCR at 1 year |
|  | Patel et al.  2025 (144) | Case–control study  (22 PCa patients  [7 aggressive and 1 indolent]) | **5-hydroxymethylcytosine (5hmC):** Genes CCDC122, NUDT15, BCCIP and KLK10 with **l**ower levels (Risk) | Sequencing analysis with 5hmC-enriched DNA | Aggressiveness  (GS ≥ 4+3) |
|  |  |  | **5-hydroxymethylcytosine (5hmC):** Genes PVT1, TRMT12, RPL30, UBR5, COX6C and ARMC2 with higher levels (Risk) |  |  |
| Jamaica | Elson et al.  2011 (109) | Case–control study  (81 PCa/ 81 controls) | **DARC** negativity (NSS) | PCR | Aggressiveness  (GS 7–10, stage T3–T4 or PSA >20ng/ml) |
|  | Gaston et al.  2024 (149) | Cohort study  (31 patients with PCa) | **West African ancestry** among Jamaican men (NSS) | Illumina Global Screening Array | Aggressiveness  (Higher pathological GG) |
| Colombia | Acosta-Vega et al.  2024 (153) | Cohort study  (230 PCa patients who underwent RP) | **Genetic ancestry**: Proportion of European, African, and Indigenous American ancestry, admixture (NSS) | Multiplex SNP genotyping via Sequenom iPLEX | Aggressiveness  (Higher GS) and BCR |
| Mexico | Arámbula-Meraz E et al. (110)  2020 | Case–control study  (13 PCa/6 controls with benign prostatic diseases) | ***PCA3:*** TAAA tandem repeats (NSS) | RT-PCR and fragment analysis | Aggressiveness  (Higher GS) |

Potential biomarkers associated with PCa prognosis, grouped by functional group, country and biomarker similarity. **Abbreviations.** PCa: Prostate cancer. SNP: Single nucleotide polymorphisms. NSS: Not statistically significant. DNA: Deoxyribonucleic acid. RNA: Ribonucleic acid. PCR: Polymerase chain reaction. RP: Radical prostatectomy. GS: Gleason score. BCR: Biochemical recurrence. BCRFS: Biochemical relapse-free survival. ISUP: International Society of Urological Pathology. BPH: Benign prostatic hyperplasia. RT-PCR: Reverse transcription polymerase chain reaction. RFLP: Restriction fragment length polymorphism. FISH: Fluorescence in situ hybridization. SSP: Sequence-specific primers.

**Supplementary table 5. Potential biomarkers associated with PCa treatment in Latin America and the Caribbean**

| **Country** | **Study Ref.** | **Study type (number of individuals)** | **Biomarker or target** | **Intervention** | **Molecular method** | **Finding (p)** |
| --- | --- | --- | --- | --- | --- | --- |
| Brazil | Magnani et al.  2012 (158) | Experimental study (30 patients with advanced PCa treated with ADT) | CD3+, CD4+ and CD8+ T lymphocytes and CCR5 chemokine receptor | Carboxymethyl-glucan from S. cerevisiae | Cytometry flow | Increase of CD3+, CD4+ and CD8+ in patients with wild-type CCR5 genotype (≤0.05). |
|  | Magnani et al.  2011 (157) | Experimental study (20 patients with advanced PCa treated with goserelin acetate) | DNA damage | Carboxymethyl-glucan from S. cerevisiae | Comet assay | Reduction in DNA damage scores of 59% on average after treatment (≤0.05) |
|  | Berger et al.  2007 (159) | Phase I clinical trial (11 patients with PCa) | DHT reaction | Autologous tumor cell vaccine and Bacille Calmette-Guérin | N/A | There was low toxicity, and it could induce cellular immune response (N/A) |
|  | Gongora et al.  2022 (160) | Case report (2 patients with mCRPC, treated with BAT & nivolumab, and radium-223 & sipuleucel-T, respectively) | CDK12 mutation | Immunotherapy and DNA-damaging therapies | Next-generation sequencing (FoundationOne) | The combination of therapies could be useful for the treatment of CDK12-mutated advanced PCa (N/A) |
|  | Ferreira Bruzzi Porto et al.  2024 (164) | Case report  (1 patient with treatment-emergent neuroendocrine PCa) | ATM mutation (3077+1G>A and 8011-1G>A), SF3B1 (G742D) mutation and intermediary tumor burden (TMB: 9 Muts/Mb) | Combined immunotherapy (ipilimumab and nivolumab) | Next-generation sequencing (FoundationOne) | Combined immunotherapy could have therapeutic potential for the treatment of combined mutations PCa, like ATM and SF3B1 |
|  | Isaacsson Velho et al.  2024 (165) | Phase II clinical trial  (38 patients with mCRPC) | DNA repair defects (unspecified) | Nivolumab | Sequencing of ctDNA | No statistically significant differences were observed in PSA50 response, objective response rate, PSA progression-free survival, or radiographic progression-free survival between patients with or without DNA repair defects |
| México | Rojas-Martinez et al.  2013 (156) | Phase I-II clinical trial (9 PCa patients who underwent radical prostatectomy) | Inflammatory cells (detection of CD4+, CD8+ and TIA-1) infiltration | AdV-tk (Herpes virus thymidine-kinase gene) + gancyclovir/valacyclovir | Immunohistochemical analysis | Neoadjuvant AdV-tk was safe and it could stimulate anti-tumor immune response (N/A) |
| Argentina, Colombia, México | Pacheco-Orozco et al.  2020 (161) | Hypothetical cost-saving analysis (N/A) | Androgen receptor splice variant 7 (AR-V7) | Abiraterone/enzalutamide vs. taxane chemotherapy | Polymerase chain reaction | The use of AR-V7 testing could improve the choice of treatment and reduce the cost associated with the management of mCRPC (N/A) |
| Chile | Reyes et al.  2013 (162) | Phase I clinical trial (20 patients with CRPC) | CD8+ IFN-γ+ T cell population and DHT reaction | Tumor antigen-presenting cells | Cytometry flow and immunohistochemical analysis | Treatment was safe, induced memory T-cell responses in vitro and in vivo (N/A) |
| Cuba | Campal-Espinosa et al.  2023 (163) | Phase I-II clinical trial (34 patients with advanced PCa) | Anti-GnRH antibodies | Gonadotropin-releasing hormone-based therapeutic vaccine | Enzyme-linked immunosorbent assay | Treatment induced anti-GnRH serum antibody response (N/A) |

Potential biomarkers associated with PCa treatment, grouped by country. **Abbreviations.** PCa: Prostate cancer. CRPC: Castration-resistant prostate cancer. mCRPC: Metastatic castration-resistant prostate cancer. BAT: Bipolar androgen therapy. DTH: Delayed-type hypersensitivity. ADT: Androgen deprivation therapy. CCR5: C-C chemokine receptor type 5. AdV-tk: Adenovirus-based vectors expressing the thymidine kinase gene. TIA-1: T-cell intracytoplasmic antigen. AR: Androgen receptor. GnRH: gonadotropin-releasing hormone

**Supplementary table 6. Other potential biomarkers associated with PCa in Latin America and the Caribbean**

| **Country** | **Study Ref** | **Study type (number of individuals)** | **Biomarker** | **Function** | **Molecular method** |
| --- | --- | --- | --- | --- | --- |
| Argentina, Brazil, Colombia, Costa Rica, Mexico, Panama, Peru | Manneh et al.  2024 (174) | Prospective, cross-sectional, multicenter study  (387 mCRCP patients from Mexico (n=90), Colombia (n=83), Panama (n=59), Argentina (n=67), Brazil (n=36), Costa Rica (n=35) and Peru (n=17)) | 25 HRR genes (***AR, ATM, ATR, BARD1, BRCA1, BRCA2, BRIP1, CDH1, CDK12, CHEK1, CHEK2,***  ***FANCA, FANCL, HDAC2, MRE11, NBN, PALB2, PPP2R2A, PTEN, RAD51B, RAD51C, RAD51D, RAD54L, STK11 and TP53***) plus ***HOXB13 and ESR1*** | Homologous recombination repair (HRR) | HANDLE HRR NGS panel |
| Argentina,  Colombia, Peru,  Chile,  Mexico | Angel et al.  2024 (155) | Retrospective multicenter study  (349 metastatic PCa patients  [Argentina (n=164), Colombia (n=147), Peru (n=15), Chile (n=14), Mexico (n=9)] | Somatic and germline mutations (**BRCA1, BRCA2, ATM, PTEN, BRIP1, BARD1, CDK12, CHEK1, CHEK2, FANCL, PALB2, PPP2R2A, RAD51B, RAD51C, RAD51D, RAD54L, AKT, PI3K, TMPRSS2-ERG, p53, Rb**) | Genes involved in DNA damage response and tumor suppression | Multigene panel analysis |
| Brazil | Campos-Fernández et al.  2024 (168) | Cross-sectional study  (8 patients with localized PCa) | **Aptamers D4** and **R4** derived from A4: Showed binding to circulating tumor cell | Oligonucleotides with specific binding properties | Fluorescence-based flow cytometry |
|  | Ariffen et al.  2024 (169) | Cross-sectional study  (31 PCa) | ***TMPRSS2-ERG*** rearrangements / fusions, ***PTEN*** deletions, ***TMPRSS2-ERG*** gene fusion and satellite-DNA sequences (centromeric regions) | Diverse biomarkers with roles in PCa progression | FISH |
|  | Ribeiro et al. 2002 (170) | Cross-sectional study  (200 individuals) | ***AR***: Number of CAG repeats. Number of GGN repeats. StuI polymorphism.  ***SRD5A2***: Ala49Thr substitution. Val89Leu polymorphism.  ***CYP17***: MspA1 | Signaling and androgen synthesis | PCR, PCR-RFLP and ASOH |
|  | Coelho et al. 2024 (172) | Cross-sectional study  (71 PCa patients  [18 ≤ 55 years and 53 ≥ 60 years]) | **BRCA2, BRCA1, MT2C, APC, NBN, ATR, KMT2D, MLH1, CDK12, GFI1B, MSH6, KLK3, PALB2, PTEN, PARP1** and **TP53:** Germline variants found  **CHEK2, HOXB13, FOXA1 and SPOP:** Germline variants not found | Genes related to DNA damage response | DNA sequencing |
|  | Lautert-Dutra et al.  2024 (173) | Cohort study  (53 PCa patients)^a^ | ***MHC*** genes: Expression  ***CDK12:*** Mutation  Immune cell composition in the tissue microenvironment | Immune response and regulation | DNA sequencing  Flow cytometry |
|  | Cisternas et al. 2004 (175) | Case-control study  (15 PCa cases/ 15 healthy controls) | ***KRAS:*** Presence of point mutations in codon 12 (2/15 PCa patients vs. 0/15 in controls) | Encodes a GTPase involved in regulating cell proliferation, differentiation, and survival | PCR-RFLP |
|  | Freitas et al.  2024 (154) | Prospective study  (89 PCa patients) | Mutations in 16 gDDR genes **(BRCA1, BRCA2, ATM, CDK12, CHEK1, CHEK2, FANCL, PALB2, PPP2R2A, RAD51B, RAD51C, RAD51D, RAD54L, TP53, BARD1, BRIP1**) | Genes involved in DNA reparation | Next-generation sequencing |
| Mexico | Chávarri-Guerra et al.  2023 (176) | Cross-sectional study  (199 PCa patients) | **DNA repair gene** pathogenic variants (**ATM, CHEK2, BRIP1, and MUTYH**).  Pathogenic variants were more common among younger patients. | Detection and reparation of DNA damage | Multigene panel analysis |
| Colombia | Acosta-Vega et al.  2024 (153) | Cohort study  (230 PCa patients who underwent RP) | **Genetic ancestry:** Higher African ancestry was associated with a younger age at diagnosis | Correlated with PCa susceptibility | Multiplex SNP genotyping via Sequenom iPLEX |
| Martinique | Marlin et al.  2020 (171) | Cross-sectional study  (46 early-onset PCa patients) | ***HOXB13***: rs77179853 (c.853delT) | Inhibitor of hormone-activated androgen receptor signaling | DNA sequencing |

Other potential biomarkers associated with PCa, grouped by country **Notes:** a=Segment of the study. **Abbreviations.** PCa: Prostate cancer. PCR: Polymerase chain reaction. RFLP: Restriction fragment length polymorphism. SNP: Single nucleotide polymorphisms. DNA: Deoxyribonucleic acid. FISH: Fluorescence in situ hybridization. gDDR: germline DNA Damage Repair
